# Amyloid-β and α-Synuclein Immunotherapy: From Experimental Studies to Clinical Trials

**DOI:** 10.3389/fnins.2021.733857

**Published:** 2021-09-01

**Authors:** Jacqui Taryn Nimmo, Louise Kelly, Ajay Verma, Roxana O. Carare, James A. R. Nicoll, Jean-Cosme Dodart

**Affiliations:** ^1^Clinical Neurosciences, Clinical and Experimental Sciences, Faculty of Medicine, University of Southampton, Southampton, United Kingdom; ^2^Yumanity Therapeutics, Boston, MA, United States; ^3^United Neuroscience, Dublin, Ireland

**Keywords:** neurodegenerative disease, immunotherapy, animal models, amyloid-β, α-synuclein, Alzheimer’s disease, Parkinson’s disease

## Abstract

Alzheimer’s disease and Lewy body diseases are the most common causes of neurodegeneration and dementia. Amyloid-beta (Aβ) and alpha-synuclein (αSyn) are two key proteins involved in the pathogenesis of these neurodegenerative diseases. Immunotherapy aims to reduce the harmful effects of protein accumulation by neutralising toxic species and facilitating their removal. The results of the first immunisation trial against Aβ led to a small percentage of meningoencephalitis cases which revolutionised vaccine design, causing a shift in the field of immunotherapy from active to passive immunisation. While the vast majority of immunotherapies have been developed for Aβ and tested in Alzheimer’s disease, the field has progressed to targeting other proteins including αSyn. Despite showing some remarkable results in animal models, immunotherapies have largely failed final stages of clinical trials to date, with the exception of Aducanumab recently licenced in the US by the FDA. Neuropathological findings translate quite effectively from animal models to human trials, however, cognitive and functional outcome measures do not. The apparent lack of translation of experimental studies to clinical trials suggests that we are not obtaining a full representation of the effects of immunotherapies from animal studies. Here we provide a background understanding to the key concepts and challenges involved in therapeutic design. This review further provides a comprehensive comparison between experimental and clinical studies in Aβ and αSyn immunotherapy and aims to determine the possible reasons for the disconnection in their outcomes.

## Introduction

From the development of the first vaccine against smallpox in 1796, immunisation has led to the worldwide reduction and eradication of numerous diseases. Over the last 30 years, immunisation has advanced beyond the treatment of infectious diseases to applications within cancer therapy and neurodegenerative disease (Jing et al., 2016; Panza et al., 2019; Zella et al., 2019; Plotkin and Cashman, 2020).

### Vaccine Designs and Challenges

Immunity requires both an innate and adaptive immune response. Innate immunity involves recruitment of resident immune cells, phagocytosis and presentation of antigen on major histone compatibility complexes (MHC), cytokine secretion and complement activation. In the CNS, the innate cells are mainly microglia. Adaptive immunity involves activation of effector and memory B-cells and T-cells for long-term immunity. B-cells provide humoral immunity by secreting high-affinity antigen-specific antibodies. Activated T-cells differentiate mainly into cytotoxic Tc-cells (CD8^+^), which induce killing of the infected cell, or helper Th-cells (CD4^+^) which coordinate the type of immune response. Th1-cells promote a pro-inflammatory environment whereas Th2-cells are anti-inflammatory (Di Pasquale et al., 2015). This means that obtaining the right balance between Th1 and Th2 responses is important in vaccine design.

Vaccine design has progressed from traditional inactivated/attenuated pathogens to elicit a more targeted antibody response using subunit, toxoid, recombinant, mRNA and peptide vaccines. In neurodegenerative diseases, the main challenge in vaccine design is overcoming immune tolerance to self-antigens while avoiding autoimmunity. This can partly be remedied by designing vaccines that selectively target misfolded protein conformations and not the healthy protein, and immunotherapies that have incorporated such designs have been reviewed in detail (Plotkin and Cashman, 2020). Immune tolerance can be overcome by using fusion peptides of self and non-self antigens or an immunogenic compound [such as keyhole limpet hemocyanin (KLH)]. The effectiveness of a vaccine depends on its ability to elicit a potent immune response, which is also influenced by the age related decline in immune competency and results in reduced production of antibodies on exposure to antigen (Grubeck-Loebenstein et al., 1998; Saurwein-Teissl et al., 2002). To overcome the lack of antigen immunogenicity, adjuvants are incorporated into the vaccine to enhance the immune response.

### The Role of Adjuvants in Immunotherapy

Adjuvants initiate a rapid, local, antigen-independent response. Attenuated/inactivated vaccines contain endogenous adjuvants, however, vaccines containing purified antigen do not and require adjuvants to enhance the immune response. The mechanism of action of adjuvants is not completely understood, however, they are known to upregulate chemokines, which recruit innate immune cells to the site of injection. They increase the uptake of antigen by antigen-presenting cells and MHCII presentation of antigen for T-cell activation. Adjuvants play an important part in vaccine design as they direct the type of Th-cell response and can drive the type of immune response accordingly (Korsholm et al., 2010; Awate et al., 2013).

Insoluble aluminium salts, such as Alum (potassium aluminium sulphate), Anhydrogel (aluminium hydroxide) or Adju-phos (aluminium phosphate) are the most common adjuvants used. They favour a Th2-cell response and inhibit Th1-cell responses by promoting IL-4 secretion (Marrack et al., 2009). QS-21 is a saponin purified from the *Quillaja saponaria* plant and is one of the most potent adjuvants known, stimulating both Th1 and Th2 responses (Lacaille-Dubois, 2019). *Cytosine phosphoguanine* (CpG) oligonucleotides are short synthetic segments of single-stranded DNA with unmethylated CpG motifs. They mimic bacterial antigens as unmethylated CG dinucleotides are uncommon in eukaryotes (Jabbari and Bernardi, 2004; Bode et al., 2011). CpG promotes a Th1 response, activation of cytotoxic CD8^+^ T-cells and IFN-γ production (Bode et al., 2011).

### Adapting the T-Cell Response

T-cell responses are typically directed against a small number of dominant peptide epitopes. Activation of CD4^+^ and CD8^+^ T-cells requires the engagement of T-cell receptors (TCRs) with an antigenic peptide. T-cells only recognise antigen in association with MHC. MHC-I binds endogenous antigens and are recognised by CD8^+^ TCRs, whereas exogenous antigens are presented on MHC-II which are recognised by CD4^+^ TCRs. Therefore, the binding of the antigenic peptide to MHC molecules determines the type of T-cell response. Generally, MHC-II molecules bind to peptides 12–15 amino acids in length, however, in some instances, potent peptides 3–5 amino acids, such as those derived from influenza, can trigger a T-cell response.

## Amyloid Beta Targeted Immunotherapy

Table 1 summarises the clinical trials in AD.

**TABLE 1:** Summary of Clinical trials for passive and active immunotherapy in Alzheimer’s disease.

	Phase I		Phase II		Phase III			
			
	NCT number (participants)	Study duration	NCT number (participants)	Study duration	NCT number (participants)	Study Title	Study duration	Locations
AN1792			NCT00021723 (375)	2001–2003				
CAD106	NCT00411580 (58)	2008	NCT00795418 (31)	2008	NCT02565511 (480)	Generation S1	2015–2020	United States, Canada, Europe, United Kingdom
			NCT00733863 (27)	2008				
			NCT00956410 (21)	2009–2011				
			NCT01023685 (24)	2009–2012				
			NCT01097096 (177)	2010–2012				
ACC01			NCT00498602 (160)	2007				
			NCT00479557 (86)	2007–2013				
			NCT00752232 (40)	2008–2012				
			NCT00960531 (160)	2009–2013				
			NCT00955409 (160)	2009–2013				
			NCT00959192 (32)	2009–2013				
			NCT01238991 (53)	2010–2013				
			NCT01284387 (126)	2011–2014				
			NCT01227564 (63)	2011–2014				
AD02	NCT01093664 (20)	2009–2010	NCT01117818 (335)	2010–2013				
	NCT00633841 (24)	2008–2009	NCT02008513 (194)	2013–2014				
	NCT00711321 (23)	2008–2010						
GV1001			NCT03184467 (96)	2017–2019				
			NCT03959553 (90)	2019–2022				
MEDI-1814	NCT02036645 (77)	2015–2016						
SAR-228810	NCT01485302 (48)	2012–2015						
Ponezumab	NCT00455000 (37)	2007–2009	NCT00722046 (198)	2008–2011				
	NCT00607308 (20)	2008–2010	NCT00945672 (36)	2009–2011				
	NCT00733642 (15)	2008–2009	NCT01821118 (36)	2013–2015				
	NCT01005862 (17)	2010–2012						
	NCT01125631 (8)	2010–2011						
Bapineuzumab	NCT00397891 (80)	2006–2010	NCT00112073 (234)	2005–2008	NCT00575055 (1121)		2007–2012	United States, Canada, Europe
			NCT00174525	2005–2008	NCT00574132 (1331)		2007–2012	United States, Canada, Europe
			NCT00606476 (194)	2006–2012	NCT00676143 (1100)		2008–2012	United States, Australia, Europe, Japan, United Kingdom, South Africa
			NCT00663026 (79)	2008–2010	NCT00667810 (901)		2008–2013	United States, Australia, Canada, Europe, Japan, United Kingdom
			NCT00916617 (62)	2009–2012	NCT00998764 (494)		2009–2012	United States, Australia, Europe, Japan, United Kingdom, South Africa
			NCT01254773 (146)	2010–2013	NCT00996918 (198)		2009–2012	Australia, Europe, Japan, United Kingdom, South Africa
					NCT00937352 (896)		2009–2012	United States, Canada, Europe
Solanezumab	NCT02614131 (50)	2015–2016	NCT00329082 (25)	2006–2008	NCT00905372 (1000)	EXPEDITION	2009–2012	United States, Canada, Japan
			NCT00749216 (33)	2008–2009	NCT00904683 (1040)	EXPEDITION2	2009–2012	United States, Australia, Europe, Asia, United Kingdom
			NCT01148498 (55)	2010–212	NCT01127633 (1457)	EXPEDITION EXT	2010–2017	United States, Australia, Canada, Europe, Japan, Asia, United Kingdom
			NCT01760005 (490)	2012–2022	NCT01900665 (2129)	EXPEDITION 3	2013–2017	United States, Australia, Canada, Europe, Japan, United Kingdom
			NCT04623242 (194)	2012–2020	NCT02008357 (1150)	A4	2014–2022	United States, Australia
					NCT02760602 (26)	ExpeditionPRO	2016–2017	United States, Canada, Europe, Japan, Asia, United Kingdom
Donanemab	NCT02624778(61)	2015–2019	NCT03367403(266)	2017–2021	NCT04437511 (1500)	TRAILBLAZER–ALZ	2020–2023	United States, Australia, Canada, Europe, Japan, United Kingdom
	NCT01837641 (100)	2013–2016	NCT04640077 (100)	2020–2023				
Crenezumab	NCT02427243 (60)	2015	NCT01723826 (360)	2012–2017	NCT02670083 (813)	CREAD	2016–2019	United States, Australia, Canada, Europe, Asia, United Kingdom
	NCT02353598 (77)	2015–2019	NCT01998841 (252)	2013–2022	NCT03114657 (806)	CREAD 2	2017–2019	United States, Australia, Canada, Europe, Japan, Asia, United Kingdom
			NCT03977584 (150)	2019–2022	NCT03491150 (149)	CREAD OLE	2018–2019	United States, Australia, Canada, Europe, Japan, United Kingdom
Gantenerumab	NCT03236844 (114)	2017	NCT01760005 (490)	2012–2022	NCT03444870 (1016)		2018–2023	United States, Australia, Canada, Europe, Asia
	NCT02882009 (48)	2016–2017	NCT04592341 (150)	2020–2024	NCT02051608 (389)		2014–2021	United States, Australia, Canada, Europe, Japan, Asia, United Kingdom
	NCT02711423 (18)	2016			NCT01224106 (799)		2010–2020	United States, Australia, Canada, Europe, Asia, United Kingdom
	NCT02133937 (31)	2014			NCT04339413 (116)		2020–2023	United States, Australia, Canada, Europe, Japan, Asia, United Kingdom
	NCT01636531 (120)	2010			NCT03443973 (982)		2018–2023	United States, Australia, Europe, Japan, Asia, United Kingdom
	NCT00531804 (60)	2006–2010			NCT04374253 (2032)		2021–2024	United States, Australia, Canada, Europe, Japan, Asia, United Kingdom
	NCT01656525(28)	2012–2014						
Lecanemab	NCT01230853 (80)	2010–2013	NCT01767311 (856)	2012–2025	NCT03887455 (1766)	Clarity AD	2019–2024	United States, Australia, Canada, China, Europe, Japan, Asia, Sweden, United Kingdom
	NCT02094729 (26)	2013–2015			NCT04468659 (1400)	AHEAD 3–45	2020–2027	United States, Australia, Canada, Japan, Asia, United Kingdom
Aducanumab	NCT01677572 (197)	2012–2019	NCT03639987 (52)	2018–2019	NCT02484547 (1638)	EMERGE	2015–2019	United States, Canada, Europe, Japan
	NCT01397539 (53)	2011–2013			NCT02477800 (1647)	ENGAGE	2015–2019	United States, Australia, Canada, Europe, Japan, United Kingdom
	NCT02782975 (28)	2016			NCT04241068 (2400)		2020–2023	United States, Australia, Canada, Europe, Japan, United Kingdom
	NCT02434718 (21)	2015						
UB-311	NCT00965588 (19)	2009–2011	NCT02551809 (43)	2015–2018				
			NCT03531710 (34)	2018–2019				

### Elan Pharmaceuticals: AN1792

AN1792 was developed by Elan Pharmaceuticals and was the first vaccine for treating neurodegenerative diseases. AN1792 consisted of a synthetic peptide of human Aβ_1__–__4__2_ formulated with QS-21 (Gilman et al., 2005). The resulting antibodies from immunised patients mainly targeted amino acids 1–8 of Aβ_1__–__4__2_ and were not conformation or aggregation specific (Lee et al., 2005). There was no cross-reactivity with APP protein (Lee et al., 2005).

#### Preclinical Studies in Mice

AN1792 was found to essentially prevent the onset of amyloid-β (Aβ) related AD pathology in 6 week old PDAPP mice which overexpress mutant human APP and also reduce the progression and severity of plaque formation and associated dystrophic neurites in older 11 month old mice (Schenk et al., 1999). The effect of immunisation was dependent on the levels of antibody produced (Schenk et al., 1999).

#### Clinical Trials

AN1792 was investigated in phase 1 (United Kingdom) and phase 2 (United States, Europe) trials with 3–4 year long-term follow-up of clinical outcome. The effects of immunotherapy on neuropathology was examined post mortem (section “Case Studies”). Patients were diagnosed with probable and mild-moderate AD based on the National Institute of Neurological and Communicative Disorders and Stroke–Alzheimer’s Disease and Related Disorders Association (NINCDS-ADRDA) and mini-mental state examination (MMSE 14–26) (Holmes et al., 2008). AD patients received AN1792 (50 or 225 μg) with QS-21 (50 or 100 μg) from which 23% had positive anti-AN1792 antibody titres. An extension study in 62% patients used a modified formulation of AN1792 by replacing QS-21 with polysorbate-80, which increased the antibody titre response to 59% (Bayer et al., 2005; Holmes et al., 2008). AN1792 had no effect on cognition, however, Disability Assessment of Dementia (DAD) scores showed a positive treatment effect at the final time-point week 84 (Bayer et al., 2005; Holmes et al., 2008). Treatment related adverse events (TRAEs) occurred in 24% of patients (Bayer et al., 2005).

A phase II trial was conducted in 372 patients in which AD patients received 5 intramuscular (i.m.) injections (3 months apart) of 225 μg AN1792/50 μg QS21 (Orgogozo et al., 2003). 18 patients (6%) developed meningoencephalitis, although there was no evidence of viruses or bacteria in the brain (Orgogozo et al., 2003). Patients presented magnetic resonance imaging (MRI) abnormalities and clinical symptoms thought similar to those associated with acute disseminated encephalomyelitis or meningoencephalomyelitis, which has occurred after measles vaccinations (Orgogozo et al., 2003). Seventy five percent of these patients had elevated anti-AN1792 IgG titres in the cerebrospinal fluid (CSF) and serum, although this was not correlated to the occurrence or severity of this side effect (Orgogozo et al., 2003). Sixty six percent patients recovered close to baseline status within weeks after withdrawal from the drug (Orgogozo et al., 2003). In retrospect this side effect was what is now termed ARIA (Sperling et al., 2012).

### Amyloid Related Imaging Abnormalities

A consequence of amyloid-β immunotherapy in the brain is the occurrence of vasogenic edema (VE) or microhaemorrhages, which are associated with the vascular amyloid. This is observed in MRI as abnormal hyperintensity regions and is referred to as amyloid-related imaging abnormalities (ARIA) (Sperling et al., 2011). ARIA-E describes MRI findings related to VE and ARIA-H describes cerebral microhaemorrhage (Sperling et al., 2011). ARIA can occur asymptomatically, however, typical symptoms include headache, confusion and encephalopathy (Carlson et al., 2016). Risk factors for ARIA include the presence of Apolipoprotein E ε4 (APOE4) allele which is also associated with increased vascular amyloid.

#### Case Studies

**FIGURE 1:**
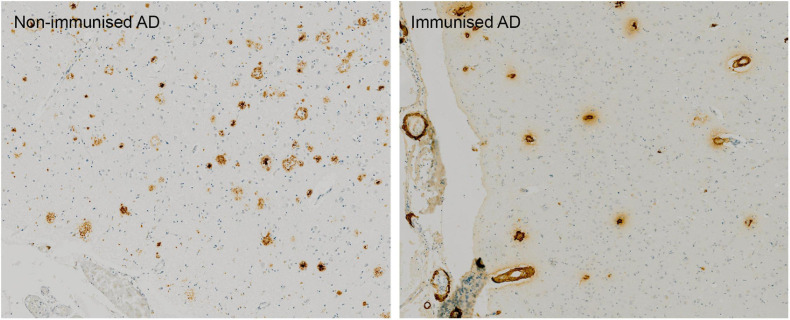
Absence of Aβ plaques and persistence of vascular CAA in the parietal neocortex of AN1792 immunised case compared to unimmunised control.

Post mortem neuropathological analysis was conducted up to a 15 years follow-up period in over 20 immunised and non-immunised cases (Nicoll et al., 2019; Boche and Nicoll, 2020). These studies revealed that at least 23% participants had alternative causes of dementia to AD (Nicoll et al., 2019), which likely affected treatment outcome. Immunisation caused a reduction in amyloid plaques that correlated with antibody titres. Tau pathology was reduced in areas cleared of amyloid plaques, which correlated with a 67–80% decrease in the tau kinase, GSK3β (Amin et al., 2015; Nicoll et al., 2019){Amin et al., 2015, Effect of amyloid-beta (Abeta) immunization on hyperphosphorylated tau: a potential role for glycogen synthase kinase (GSK)-3beta;JAR, 2019 #6877}. Immunotherapy did not prevent the spread of tau through the brain as evidenced by progression from Braak stage III–V to V–VI (Boche and Nicoll, 2020). Immunisation resulted in a 14-fold increase in cerebral amyloid angiopathy (CAA) compared to controls (Figure 1) and was accompanied by a higher density of microhaemorrhages (Boche et al., 2008). Long-term follow-up showed that AD patients could remain plaque free for up to 14 years post immunisation, and Aβ can be cleared from the vasculature despite an initial increase in CAA (Boche et al., 2008; Nicoll et al., 2019). AN1792-induced plaque removal was associated with clustering of HLA-DR^+^ and CD68^+^ microglia around plaques which was reduced after plaque removal including CD32 and CD64, but not complement (C1q) (Zotova et al., 2011, 2013). Levels of Iba1 and the number of microglia were not altered after immunotherapy and showed a variable pattern of distribution (Zotova et al., 2013). This suggested that immunotherapy alters the functional state of microglia, but not their proliferation (Zotova et al., 2013).

Post-mortem examination of two meningoencephalitis cases showed similar results regarding amyloid plaque load, tau pathology and microglial activation. Decreased Aβ burden was accompanied by a decrease in the oxidative stress markers SOD-1 and SAPK/JNK as well as P38 tau kinase (Nicoll et al., 2003; Ferrer et al., 2004). The inflammatory response that resulted in meningoencephalitis was associated with infiltration of microglia and CD4^+^/CD8^+^ T-cells (Marciani, 2016). Marciani (2016) suggested that this could be attributed to an imbalance of Th1/Th2 activation that was induced by QS-21 and further amplified by polysorbate-80 (Marciani, 2016). The QS-21 adjuvant is biased toward a Th1 pro-inflammatory response which drives cellular immunity to destroy infected cells, and resulted in a subset of auto-aggressive T-cells and meningoencephalitis. This is consistent with the observation from both Nicoll et al. (2003) and Ferrer et al. (2004) that infiltration of T-cells was largely CD4^+^T-cells. Other studies using QS-21 had similar effects and clinical trials were discontinued (Arai et al., 2015).

These observations from the AN1792 clinical trials have provided proof of principle that, remarkably, the pathology of AD can be altered by Aβ immunotherapy, also raising the prospect that other neurodegeneration-associated protein aggregates could be removed by immunotherapy. Specifically, AD plaques can be removed and this is associated with reductions in aggregated tau. Although AN1792 did not progress because of inflammatory side effects, these studies have informed the design of vaccines firstly to avoid harmful Th1 pro-inflammatory responses and secondly to better understand how mobilising plaque amyloid increases CAA and leads to ARIA most likely due to burdening the intramural periarterial drainage system. Subsequent development of vaccines has aimed at using short peptides of Aβ_1__–__4__2_ that contain B-cell, but not T-cell specific epitopes. However, in order to reproduce the conformational epitopes found in natural immunogens that induce natural protective immunity, both B- and T- cell epitopes will be required. Importantly, a combination of immunogen with Th2 adjuvants that are biased toward a systemic Th2 anti-inflammatory response are essential to elicit an immune response that mimics the natural protective immunity and avoids harmful side effects. The inflammatory complications of AN1792 prompted a shift away from active to passive vaccines of which Bapineuzumab was the first.

### Passive Immunotherapy

Characteristics of passive immunotherapies are summarised in Table 2.

**TABLE 2:** Characteristics of passive immunotherapy for Alzheimer’s disease in phase 3.

	Bapineuzumab	Solanezumab	Donanemab	Crenezumab	Gantenerumab	Lecanemab	Aducanumab
Epitope	Aβ_1__–__5_	Aβ_1__6__–__2__6_	Aβ(p3–42)	Aβ_1__6__–__2__4_	N-terminal Aβ	Arctic mutation of Aβ42	Aβ_3__–__6_
Isotype	Humanised IgG1	Humanised IgG1	Humanised IgG1	Humanised IgG4	Human IgG1	Humanised IgG1	Human IgG1
Specificity	High affinity for Monomeric Aβ Fibrillary Aβ Plaques	Monomeric Aβ Fibrillary Aβ Plaques Aβp3–42	Plaques	High affinity for oligomers, also recognises Aβp3–42	High affinity for fibrillar and aggregated Aβ	Protofibrils	High affinity for fibrillar and aggregated Aβ
Route of administration	IV	IV	IV	IV	SC	IV	IV
Dose at phase 3	0.5–1.0 mg/kg (6 shots 12 w apart)	400 mg (4 w apart up to 2 year)	10 mg/kg (4 shots 4 w apart)	15 mg/kg (24 shots 4 w apart)	105, 225, 1,200 mg (25 shots 4 w apart)	10 mg/kg (36 shots 2 w apart)	10 mg/kg (25 shots 4 w apart)
**Primary outcome**							
Parent study	(ADAS-Cog)/11 and DAD w78	ADAS-Cog14 w80	iADRS, CDR-SB, ADAS-Cog13	CDR-SB w77-105	ADAS-Cog13 w104, CDR-SOB w116	CDR-SB 18 months, PACC5 and PET W216	CDR-SB w78
Extension study	SAE w195	SAE w104	N/A	SAE w54	SAE w104	N/A	SAE w118

*IV, intravenous; SC, subcutaneous; SAE, serious adverse events.*

#### Janssen/Pfizer: Bapineuzumab

Bapineuzumab was the first humanised monoclonal antibody for AD. It was designed against Aβ_1__–__5_ such that it does not recognise N-truncated or modified Aβ (Bouter et al., 2015).

##### Preclinical Studies in Mice

Preclinical studies used the murine version of Bapineuzumab, 3D6, in transgenic PDAPP mice. Pharmacokinetics were investigated at extremely low doses by radiolabelling the antibody (1μCi ^125^I-3D6) (Bard et al., 2012). After a single intraperitoneal (i.p.) injection into 16 month PDAPP mice, ^125^I-3D6 accumulated only in plaque rich regions of the brain (hippocampus and cortex), and was absent in WT mice (Bard et al., 2012). ^125^I-3D6 accumulation correlated with age as mice accumulated more plaques with time. ^125^I-3D6 radioactivity in the brain was sustained for over 27 days, suggesting that it remains bound to Aβ over prolonged periods (Bard et al., 2012).

Another study treated 12–18 months old PDAPP mice with 3D6, which effectively cleared amyloid deposits within the vasculature (Zago et al., 2013). PDAPP mice also develop CAA similar to that observed in AD patients. CAA was cleared over time (9 months) with weekly 3D6 infusions, but this induced transient increases in microhaemorrhages and capillary Aβ as parenchymal amyloid is cleared along intramural periarterial drainage routes (Zago et al., 2013). Microhaemorrhages increased after 7–24 injections which then decreased back to baseline levels by the 36th dose (Zago et al., 2013). This is reflected in clinical trials where the occurrence of microhaemorrhages increases on commencement of immunotherapy and decreases upon multiple doses. The mechanism for the microhaemorrhage was thought to involve the exposure of damaged vessel walls due to removal of amyloid. Cerebral blood vessels in PDAPP mice show degeneration in smooth muscle actin (SMA) in the presence of Aβ deposits and increase variance in SMA and basement membrane (ColIV) (Zago et al., 2013). Prior to the immunotherapy-related increase in microhaemorrhage, the thickness of SMA and ColIV was increased (Zago et al., 2013). Despite this, the uniformity of vessel wall components was restored to levels found in non-transgenic mice after prolonged 3D6 treatment, including blood vessels that had previously demonstrated microhaemorrhages (Zago et al., 2013). Variability in basement membrane thickness was restored faster (12 weeks) than smooth muscle cells (36 weeks) (Zago et al., 2013). Being a passive immunisation, it was unlikely that 3D6 would induce a cellular immune response. In accordance with this, no proliferative T-cell response to Aβ exposure was observed in splenocytes after 6 months of treatment (Bard et al., 2000). However, microglia became activated to a phagocytic phenotype through Fc receptor engagement (Bard et al., 2000). In an *ex vivo* assay, 3D6 treatment induced phagocytic clearance of amyloid plaques in AD human and PDAPP mouse brain sections that had been cultured with primary microglia for 24 h (Bard et al., 2000). Despite the effective clearance of amyloid plaques and the potential recovery of vascular damage, these studies did not conduct behavioural tests to analyse the effect of immunotherapy on cognition.

##### Clinical Trial

Bapineuzumab entered an 8 month phase 2 multiple ascending dose study to test the safety and efficacy in AD patients (Salloway et al., 2009). The study enrolled 234 participants (APOE4 carriers and non-carriers) with MMSE and Rosen Hachinski Ischemic scores, and MRI scans indicative of mild-moderate AD. Patients received 6 infusions of Bapineuzumab (13 weeks apart) of four doses from 0.15 to 2.0 mg/kg and placebo. After 78 weeks significant differences were observed in ADAS-Cog cognitive scores when the four dose cohorts were combined (Salloway et al., 2009). DAD and MMSE tests showed a trend toward improvement in function and cognition between 50 and 78 weeks. Correlating with this, the CSF biomarker phosoho-tau-181 showed a decreasing trend with Bapineuzumab treatment, however, no difference was observed in Aβ at 78 weeks (Salloway et al., 2009). Despite these seemingly promising results, MRI analysis revealed a dose-dependent increase in the occurrence of VE up to 26.7% with the highest dose. VE also increased with APOE4 copy number, which is likely due to the greater extent of CAA in APOE4 carriers (Salloway et al., 2009). The MRI abnormalities resolved several months after termination of Bapineuzumab administration while symptoms improved after a few weeks (Salloway et al., 2009).

The potential treatment effects of Bapineuzumab led to four phase 3 trials and an extension study (Salloway et al., 2014; Vandenberghe et al., 2016). These studies included APOE4 carriers and non-carriers and used the same treatment strategy as the phase 2 trial, omitting the 2.0 mg/kg dose due to high rate of ARIA. In contrast to the phase 2 trial, there was no treatment effect of Bapineuzumab on cognitive outcome compared to placebo in all phase 3 trials. At the final time point, the 0.5 mg/kg APOE4 non-carrier group showed a tendency toward improved DAD (Salloway et al., 2014) in European and American cohort and ADAS-Cog11 (Vandenberghe et al., 2016) score in the Japanese cohort. Although this trend was inconsistent between studies, it suggested a delayed response in which longer exposure to Bapineuzumab may improve cognitive decline, however, a 1 year extension study showed no change in scores from the parent study (Ivanoiu et al., 2016).

No difference in amyloid clearance was recorded in standardised value uptake ratio (SUVR) for Pittsburgh compound B positron emission tomography (PIB-PET) in APOE4 compared to placebo (Salloway et al., 2014). It is notable, however, that while SUVR increased over 71 weeks in placebo, Bapineuzumab treated APOE4 patients remained steady at baseline levels, suggesting a possible decreased rate of amyloid accumulation. SUVR levels were more variable in non-carriers and showed no significant difference from baseline. At 71 weeks a trend to decrease in SUVR could be seen at 1.0 mg/kg cohort compared to placebo (Vandenberghe et al., 2016). This may have been due to the small patient cohort in this group (*n* = 12–27) but the notable decrease observed suggests that a significant effect may occur at a later time point—this was not measured in the extension study. Phospho-tau levels in CSF samples from 76 to 138 APOE4 carriers showed a treatment related decrease with Bapineuzumab (Salloway et al., 2014). In non-carriers, only a trend in decreasing p-tau was observed at higher doses (Vandenberghe et al., 2016). Only 14–15 patients continued CSF sampling in the extension study and this showed no significant change from baseline values. Bapineuzumab did not alter the annual rate of brain volume loss of 18 ml/year (Vandenberghe et al., 2016), measured by vMRI.

The main treatment-related adverse effect (TRAE) was ARIA-E and microhaemorrhage, which limited use of higher doses potentially hindering its efficacy. ARIA-E was 15% higher in Bapineuzumab treated APOE4 carriers than placebo and led to 3% patients discontinuing the study (Vandenberghe et al., 2016). The occurrence of ARIA-E increased with dose in non-carriers from 4% at 0.5 mg/kg to 14% higher than placebo at 1.0 mg/kg (Salloway et al., 2014). In addition intracranial haemorrhage, seizure, deep vein thrombosis and pulmonary embolism were more frequent with Bapineuzumab treatment in APOE4 compared to placebo (Salloway et al., 2014; Vandenberghe et al., 2016). In the extension study, APOE4 carrier patients continuing on Bapineuzumab showed a 4% reduction in TRAE and SAE compared to patients previously on placebo (Ivanoiu et al., 2016). In non-carriers there was an overall dose-dependent decrease in TRAE in patients who were on Bapineuzumab in the parent study compared to patients on placebo (64–73%). However, ARIA-E occurrence increased with patients previously on Bapineuzumab. The extension study did not include a placebo cohort as placebo patients from the parent study were put on Bapineuzumab therapy, therefore end-point measurements could not be compared to normal progression of AD. The dose dependent effects of Bapineuzumab on the occurrence of microhaemorrhages and ARIA-E is consistent with mouse studies, however, these were not always transient, but rather still occurred at similar levels in the extension study (Ivanoiu et al., 2016).

Bapineuzumab reduced amyloid as assessed with PET scanning by a small amount but it did not improve clinical outcomes in patients with Alzheimer’s disease. The doses of Bapineuzumab used in these studies were limited because of higher rates of ARIA-E at higher doses. Bapineuzumab phase 3 trials were discontinued due to lack of clinical benefit.

#### Eli Lilly: Solanezumab

Solanezumab is a humanised monoclonal antibody targeting Aβ_1__6__–__2__6_ (DeMattos et al., 2001; Bouter et al., 2015). In contrast to Bapineuzumab targeting the cerebral vasculature and increased incidence of ARIA-E, Solanezumab is selective for soluble Aβ (Zhao et al., 2017). This implies that it should not have disrupted existing plaques and so not lead to worsening of CAA (Carlson et al., 2016). Unlike Bapineuzumab, Solanezumab was able to detect N-terminally modified Aβ peptides Aβ_4__–__4__2_ and pyroglutamate Aβ_3__–__4__2_ (Bouter et al., 2015). Unexpectedly, immunohistochemical analysis in human and mouse tissue showed target engagement with plaques, CAA and intraneuronal amyloid (Bouter et al., 2015).

##### Preclinical Studies in Mice

M266 is the murine version of Solanezumab. M226 has been found to reduce Aβ in CNS by facilitating its removal from the brain to plasma. M266 was specific for soluble Aβ monomers, not oligomers, hence the greater effect of M266 on clearing the more soluble Aβ40 than Aβ42 (Mably et al., 2015).

A single i.v. injection of 500 μg M266 into young (3 month) and aged (13–22 month) PDAPP mice dramatically increased plasma antibody-Aβ complexes 24 h later compared to controls (DeMattos et al., 2001, 2002). This was correlated with amyloid burden in the hippocampus and cortex (DeMattos et al., 2002). In the CSF, M266 had a larger and more immediate effect on the increase in Aβ40 than Aβ42 in PDAPP and J20 transgenic mice (DeMattos et al., 2001; Mably et al., 2015). Since PDAPP mice only produce human Aβ in the brain, the discovery of Aβ in plasma suggests a translocation from the CNS (DeMattos et al., 2001). This was confirmed by injecting Aβ into the CSF immediately after M266 immunisation and measuring the increase in plasma levels of Aβ-M266 complexes over 4 days (DeMattos et al., 2001). Prolonged treatment in young (4 m) PDAPP mice of weekly infusions for 5 months showed little change in plaque coverage compared to controls, although the level of Aβ in brain homogenates measured by ELISA was reduced (DeMattos et al., 2001). Importantly, PDAPP mice did not have Aβ deposits even after 9 months of age, confounding the interpretation of these results (DeMattos et al., 2001). Similarly, the treatment with M266 in 9.5 month old J20 mice did not reduce Aβ in the frontal cortex or hippocampus and M266 was not found associated with plaques even after 14 weekly i.p. injections (Mably et al., 2015). M266 was also found to restore acetylcholine (ACh) neurotransmission in PDAPP mice (Bales et al., 2006). Microhaemorrhage and inflammation were analysed in 9.5 month J20 mice and showed no effect after 3 months of weekly immunisations and there was no change in markers of p-tau, APP or inflammation.

M266 immunotherapy gave conflicting results in behavioural tests. In one study using11 and 24 month old PDAPP mice, there was recovery of novel object recognition after a single dose or chronic (6 weeks) administration of M266. Improvement in hole board learning and memory task was also reported and these behavioural effects occurred without change in Aβ burden (Dodart et al., 2002). Consistent with this, another study showed that a single injection of M266 in 4–6 month PDAPP mice restored hyperactivity back to Wt levels (Bales et al., 2006). In contrast, J20 mice did not show any treatment effect of M266 in spatial memory tasks with persistent hyperactivity in the open field task and more errors in a radial arm maze compared to Wt mice. This may be due to the model used as J20 mice have a higher level of Aβ oligomers (putatively the more toxic species) compared to PDAPP and also had a 20% increase in mortality due to M266 compared to Wt and PDAPP (Mably et al., 2015).

##### Clinical Trials

Single and multiple-dose phase 2 trials were conducted in a small cohort of mild-moderate AD patients and demonstrated safety and tolerability of Solanezumab with no TRAE including microhaemorrhage or VE (Siemers et al., 2010; Farlow et al., 2012). Pharmacodynamic profile of single doses (0.5–10 mg/kg) of Solanezumab in Japanese patients with moderate AD was assessed over 112 day period (Uenaka et al., 2012). Clearance and volume of distribution was similar across doses but there was a dose-dependent increase in the magnitude and time to reach maximum concentration (Uenaka et al., 2012). Aβ_1__–__4__0_ increased in the plasma consistent with Solanezumab targeting soluble Aβ (Uenaka et al., 2012). Solanezumab was administered every week or every 4 weeks at 100 or 400 mg up to 12 infusions (Farlow et al., 2012). Total (bound and unbound) Aβ_1__–__4__0_ and Aβ_1__–__4__2_ in the plasma and CSF increased dose-dependently with little effect from dose frequency. In the CSF, unbound Aβ_1__–__4__2_ increased (indicative of plaque mobilisation) and unbound Aβ_1__–__4__0_ decreased (indicative of soluble Aβ) which is consistent with target engagement of Solanezumab to soluble Aβ_1__–__4__0_ (Farlow et al., 2012). In this phase 2 trial, no cognitive effects as measured by ADAS-Cog were recorded after administering Solanezumab for12 weeks.

Solanezumab underwent three phase 3 trials (Expedition 1–3). Results from primary and secondary outcome measures were consistent across these trials. Expedition 1 and 2 were identical in design and enrolled over 1,000 patients with mild-moderate AD based on MMSE score and NINCDS-ADRDA (Doody et al., 2014). Later it was found by ^18^florbetapir-PET imaging that 10% of clinically defined moderate AD and 25% mild AD subjects were negative for amyloid in their brain, which led to Expedition 3 using a more refined diagnosis to enrol only patients with brain amyloid (Chen et al., 2016; Honig et al., 2018). In Expedition 1 and 2 each patient received monthly 400 mg/ml doses of Solanezumab every 4 weeks for 18 months (Doody et al., 2014). Cognition was assessed over an 80 week period from start of treatment using MMSE, ADAS-Cog11 and ADAS-Cog14 (which is designed to better differentiate mild AD). At week 80, the decline in ADAS-Cog score (change from baseline) was greater in placebo compared to Solanezumab patients. Although this was not significant at week 80, in Expedition 2 and pooled data from Expedition 1–2 the difference in ADAS-Cog11 score reached significant levels at week 52 and 64 (Doody et al., 2014; Liu-Seifert et al., 2015); however, this only delayed the progression of cognitive decline by a maximum of 16 weeks. Changes in ADAS-Cog14 scores were significantly different only for mild AD patients after 64 weeks of treatment (Doody et al., 2014; Liu-Seifert et al., 2015).

The pattern of functional and cognitive treatment effects was persistent during the 3.5 year extension study. The extension lacked a placebo control cohort, as placebo patients in the parent study were then administered Solanezumab, making it difficult to confidently assess treatment effect at the later time points. Differences in cognition (ADAS-Cog14) between patients continuing on Solanezumab and placebo patients starting Solanezumab treatment were significant during the extension period up to final time point of 184 weeks (Liu-Seifert et al., 2015). Despite the variation in behavioural outcome in mouse studies, these phase 3 trials were one of the first to show favourable cognitive outcome measures for mild AD and provided support for Expedition 3 (Honig et al., 2018). No significant change in cognitive outcome was observed between placebo and Solanezumab, however, similar to Expedition 1&2, Solanezumab treatment showed marginally reduced cognitive decline over the 72 week period (Honig et al., 2018).

Treatment with Solanezumab resulted in a significant increase in plasma and CSF Aβ compared to placebo, showing high and sustained level of peripheral target engagement (Doody et al., 2014; Honig et al., 2018). There was no change in CSF tau and p-tau biomarkers or in brain volume, measured by MRI with an average of 20 cm^3^ whole brain loss and 6.7 cm^3^ ventricular enlargement by the end of the study in both placebo and Solanezumab groups (Siemers et al., 2016). Since Solanezumab does not target fibrillary Aβ, it is not surprising that SUVR did not change with ^18^F-florbetapir-PET analysis in Expedition 1&2. However, an alternate method of analysis designed to improve statistical power in smaller samples using a subject-specific white matter reference region instead of the cerebellum found a significant decrease in SUVR with Solanezumab in mild AD (Fleisher et al., 2017).

With respect to TRAEs, patients in the Solanezumab cohorts had 1.8% less vascular disorders, 0.6% less cerebral microhaemorrhages and 0.7% less ARIA-H. 0.5% more patients suffered ARIA-E after Solanezumab administration which completely or partially resolved during follow-up (Siemers et al., 2016). ARIA-E had a trend of earlier onset and longer time to resolve in Solanezumab treated groups compared to placebo (Carlson et al., 2016). The frequency of ARIA-E did not increase much during the extension study (Liu-Seifert et al., 2015). 32% of patients who developed ARIA-E were APOE4 homozygotes compared to 13% in non-APOE4 carriers consistent with the idea that APOE4 is a risk factor for ARIA-E (Carlson et al., 2016). In contrast to Bapineuzumab clinical trials which had a high, dose dependent occurrence of ARIA-E (9.7–26.7%), Solanezumab had a comparatively low occurrence of ARIA-E (1%) which is likely due to its selectively for soluble Aβ which is not associated with vascular Aβ (Carlson et al., 2016). Most of the phase 3 clinical trials for Solanezumab have been terminated due to lack of efficacy.

##### Case Study

Post mortem neuropathology was reported of a 79 years old male who completed 9 months of therapy and showed no cognitive or functional improvement, but rather progressive decline (Roher et al., 2016). While originally diagnosed as AD, depigmentation of substantia nigra coupled with unsteady gait and the presence of Lewy bodies (Roher et al., 2016) suggest that this may have been a mixed case of AD/DLB.

Compared to non-immunised (NI) AD cases, CAA in leptomeningeal arteries, arterioles and capillaries was increased by 230%. Consistent with preclinical studies in mice, Solanezumab did not alter plaque burden in the cortex or total plaque scores compared to NI-AD cases. Analysis of Aβ levels in the frontal and temporal cortices by ELISA showed an increase in Aβ40, but not Aβ42, with Solanezumab treatment (only a small increase in temporal cortex) (Roher et al., 2016) again reflecting animal studies. Soluble Aβ40 increased over 4.4-fold in frontal cortex and was much higher (80-fold) in the temporal cortex but insoluble Aβ40 did not increase as much (5.6- and 13-fold in frontal and temporal cortex, respectively) (Roher et al., 2016) consistent with Solanezumab targeting soluble Aβ. Proinflammatory cytokines TNF-α and IL1β were similar between immunised and non-immunised AD in frontal and temporal cortex (Roher et al., 2016).

#### Eli Lilly: Donanemab

Donanemab (LY3002813) is an IgG_1_ monoclonal antibody that has been humanised from mouse mE8-IgG2a. Donanemab is specific for the pyroglutamate form of Aβ(p3–42) present only in amyloid deposits and therefore aimed to remove existing plaques rather than soluble Aβ.

##### Preclinical Studies in Mice

mE8-IgG2a was administered to aged PDAPP mice (24–25 m with maximal plaque load) at 12.5 mg/kg by weekly i.p. injections for 3 months. The mE8-IgG2a antibody entered the brain and bound to plaques which was associated with microglial convergence. Immunotherapy resulted in a 53% decrease of Aβ_42_ levels in hippocampal and cortical lysate, which was confirmed by histology. No difference in plasma Aβ40/42 was observed in treated mice. In contrast to Bapineuzumab, existing plaques were removed without CAA-related microhaemorrhage (Demattos et al., 2012).

##### Clinical Trials

Donanemab completed two phase 1 trials in 61–100 participants. Patients were administered 4 monthly i.v. infusions of five different doses up to 10 mg/kg, with a 12 week follow-up period (Irizarry et al., 2016; Lowe et al., 2021). Pharmacokinetics of Donanemab showed a surprisingly short half-life of 4–10 days. Despite this, Donanemab significantly reduced amyloid load by 40–50% in PET scans at 10 mg/kg (Irizarry et al., 2016; Lowe et al., 2021). Donanemab was well tolerated at the highest dose with only 2 cases of ARIA-H.

In its first TRAILBLAZER-ALZ phase 2 trial, Donanemab met its primary endpoint with a 32% change from baseline in the Integrated Alzheimer’s Disease Rating Scale (iADRS) Score (Mintun et al., 2021). The iADRS is a combination of ADAS-Cog13 and ADCS-iADL testing both cognition and function. 266 patients with early symptomatic AD (determined by MMSE, amyloid flortaucipir PET scans and low tau levels) were given monthly injections of 1,400 mg Donanemab for 72 weeks (Mintun et al., 2021). The first three doses were given at 700 mg. There was no difference in secondary outcomes measures including CDR-SB, ADAS-Cog13, and ADCS-iADL. Amyloid loads decreased by 78%, leaving 66% of participants amyloid negative by the end of the trial. However, this also resulted in 25% ARIA-E of which 6% were symptomatic (Mintun et al., 2021). Plaque clearance did not show any evidence of reduction in global tau on PET imaging with Donanemab treatment compared to placebos.

This led to an ongoing TRAILBLAZER-ALZ2 enrolling 500 participants with the same criteria for mild-moderate AD, however, patients with more advanced tau were not excluded. The primary outcome measure in this phase 2 trial was change from baseline in CDR-SB. A follow-on study (TRAILBLAZER-EXT) has enrolled 100 patients with remaining plaques from TRAILBLAZER-ALZ with primary outcome measures of ADAS-Cog13 and ADCS-ADL.

#### AC Immune: Crenezumab

Crenezumab was first developed by AC Immune, using a SupraAntigen^TM^ platform, and was later licenced to Genentech for its manufacture and clinical development. Crenezumab is a fully humanised antibody (Bouter et al., 2015) incorporating an IgG4 isotype, which has reduced Fcγ binding affinity and hence reduced effector function of microglia and inflammation. Studies on the crystal structure of Crenezumab-Aβ complex have shown that Crenezumab recognises an extended conformation specific epitope on the mid-region of the Aβ peptide (Ultsch et al., 2016) (residues 16–24 (Zhao et al., 2017)) and can detect N-terminally modified Aβ peptides and pyroglutamate Aβ3–42 (Bouter et al., 2015). Crenezumab binds to multiple forms of Aβ with a high affinity for oligomers. On engagement with Aβ, Crenezumab prevents the formation of the β-hairpin conformation that is necessary for oligomerisation and hence it prevents Aβ aggregation as well as promotes its disaggregation (Ultsch et al., 2016).

##### Preclinical Studies in Mice

Crenezumab was generated by immunising mice with Aβ peptide using a liposomal vaccine. Resultant antibodies were selected based on their ability to bind multiple forms of Aβ and prevent oligomer assembly. The antibody was then humanised onto an IgG4 backbone as mice do not produce IgG4 antibodies (Adolfsson et al., 2012).

Although there are no preclinical behavioural studies reported with Crenezumab as far as we are aware, Crenezumab demonstrated neuroprotective properties both *in vitro* and *in vivo*. Primary cortical cultures treated with 2.5–5 μM Aβ_1__–__4__2_ oligomers over 24 h showed reduced cell viability. This was restored close to baseline levels after treatment with pre-bound Crenezumab-Aβ complexes. Another *in vitro* study demonstrated preservation of neurite branches in cortical cultures exposed to Aβ as well as prevention of neuronal Aβ uptake, after treatment with Crenezumab-Aβ complexes. The mechanism of clearance was associated with microglial phagocytosis as Aβ colocalised with Iba1 staining for microglia (Adolfsson et al., 2012). When Crenezumab (IgG4) was compared to an identical IgG1 antibody, which fully engages Fcγ receptors and activates microglia, the IgG4 induced a 6% higher cell survival in primary cortical cultures and reduced TNF-α release. When injected directly into the brains of Tg256 mice, Crenezumab did not show significant inflammatory changes after 7 days, measured by TNF-α, IL1β release and upregulation of microglial markers (CD68 and CD11b) (Fuller et al., 2015). The ability of Crenezumab to induce amyloid clearance was demonstrated by *in vivo* live imaging through cranial window in 10 month old hAPP^(V7171)^/PS1 mice which showed that after a single dose of Crenezumab plaque size decreased significantly over 3 weeks (Adolfsson et al., 2012).

##### Clinical Trials

The safety and tolerability of a single dose (0.3–10 mg/kg) or 4 weekly doses (0.5–5 mg/kg) of Crenezumab were investigated in a phase 1 multicentre trial in mild-moderate AD (determined by MMSE and National Institute of Neurological and Communicative Disorders and Stroke and the AD and Related Disorders Association criteria) (Adolfsson et al., 2012). The antibody had a half-life of 18–23 days and a dose dependent increase in Aβ plasma concentration was observed (Adolfsson et al., 2012) suggesting treatment dependent clearance from the brain. Since this initial trial, Crenezumab has completed at least two phase 2 studies (plus one ongoing phase 2 trial) and is currently under investigation in phase 2 and 3 trials in presymptomatic PSEN-1 mutation familial AD subjects in Columbia (Tariot et al., 2018).

The 73 week phase 2 trials, ABBY and BLAZE, were identical in design and conducted in the US and Europe. They included over 400 patients with mild-moderate AD. Patients received either a low dose (300 mg as 2 weekly s.c. injections) or a high dose (15 mg/kg as i.v. every 4 weeks) of Crenezumab (Cummings et al., 2018). No significant treatment effect was observed on cognition (change from baseline in ADAS-Cog12, CDR-SB, ADCS-ADL scores) in either low or high dose cohorts, although a slower rate of decline was observed with 15 mg/kg at earlier time points (week 25–49) (Cummings et al., 2018). In both phase 2 trials a notable reduction in decline was observed in a subset of mild patients at high dose, and the percentage reduction relative to placebo consistently increased in ADAS-Cog in relatively mildly affected AD patients (Cummings et al., 2018; Salloway et al., 2018). A phase 3 trial sponsored by Genentech is currently testing the hypothesis that earlier treatment and a higher dose is associated with improved outcome (CREAD 1 and 2) (Salloway et al., 2018).

A significant increase in CSF Aβ42 and plasma Aβ40&42 was observed after 68 weeks, suggesting penetration of Crenezumab into the CNS, although CSF Crenezumab and Aβ were not correlated in time (Cummings et al., 2018; Salloway et al., 2018). There was no treatment effect on CSF tau/p-tau and no change in volumetric MRI or SUVR with PET imaging (only a trend toward higher amyloid reduction was observed at higher doses) (Cummings et al., 2018; Salloway et al., 2018). In ABBY, a dose dependent increase in percentage of SAE was recorded with 0.6% patients with ARIA-E (15 mg/kg), however, Crenezumab therapy showed less ARIA-H and microhaemorrhage compared to placebo (Cummings et al., 2018).

Interim analysis of the likelihood for Crenezumab to meet its primary endpoint led to its discontinuation from clinical trials.

#### BioArctic Neuroscience and Esai: Lecanemab (BAN2401)

After discovering the Arctic APP mutation, which promotes formation of Aβ protofibrils, Lecanemab was developed from the mouse mAb158 antibody which is highly selective for protofibrils and prevented fibril formation *in vitro* (Lord et al., 2009; Magnusson et al., 2013).

##### Preclinical Studies in Mice

Systemic administration of radiolabelled mAb158 showed that it accumulated in the brain parenchyma with little association with plaques and CAA (Magnusson et al., 2013). A single shot of mAb158 (50 mg/kg) in aged Tg-ArcSwe mice caused a 40% reduction in soluble Aβ (Syvänen et al., 2018). mAb158 did not affect existing plaques but prevented the formation of new ones in young mice after 16 i.p. injections (1 week apart) at 3 mg/kg (Lord et al., 2009). There was no functional difference between Tg and Wt mice at this age so no treatment effect was observed (Lord et al., 2009).

##### Clinical Trials

After showing a favourable safety profile in two phase 1 trials, Lecanumab was tested in ongoing phase 2 trials with an adaptive Bayesian design (Satlin et al., 2016). Mild-moderate AD patients based on Wechsler Memory Scale-IV Logical Memory II (WMS-IV LMII), MMSE, PET, and CSF Aβ were administered 2.5, 5, 10 mg/kg doses biweekly or monthly for 1 year (Swanson et al., 2021). A dose dependent reduction in PET SUVR occurred leaving 80% amyloid negative at the end of treatment (Swanson et al., 2021). While total-tau levels remained unchanged, a significant increase in CSF Aβ42 and decrease in p-tau relative to placebo occurred by 18 months (Swanson et al., 2021). Significant reduction in Alzheimer’s Disease Composite Score (ADCOMS) (15–30%) and ADAS-Cog14 (47%) was observed by 18 months with 10 mg/kg Lecanumab compared to placebo (Swanson et al., 2021). A notable (not significant) decrease occurred in CDR-SB by 17–26%. Effect on cognition was greater in APOE4 subjects. The main safety finding was ARIA with 10% incidence of ARIA-E and ARIA-H which was more prominent in APOE4 carriers which resolved over 12 weeks. However, 36% Lecanumab patients were discontinued mainly due to ARIA-E (Swanson et al., 2021).

Lecanumab is currently in two phase 3 trials, CLARITY AD and AHEAD 3–45 to test the safety of 10 mg/kg dose over 18 months with change in CDR-SB, Preclinical Alzheimer Cognitive Composite 5 (PACC5) Score and PET imaging as the primary outcome measures.

#### Hoffmann-La-Roche: Gantenerumab

Gantenerumab recognises a conformational epitope that contacts the N-terminus and mid-region of the Aβ peptide and has a high affinity for fibrillary or aggregated Aβ. Gantenerumab was the first entirely human anti-Aβ monoclonal antibody to enter the clinic, in contrast to Bapineuzumab and Solanezumab, which were produced as murine antibodies and subsequently humanised (Ostrowitzki et al., 2012). This was achieved by use of the MorphoSys Hu-CAL-Fab1 phage display Human Combinatorial Antibody Library to select an antibody clone for optimisation by *in vitro* affinity maturation on fibrillar Aβ (Ostrowitzki et al., 2012). Reiterative cycles of CDR optimisation enabled the selection of an antibody with sub-nanomolar K_D_ affinity values for fibrillar and oligomeric Aβ (Bohrmann et al., 2012).

##### Preclinical Studies in Mice

The pharmacokinetic profile of Gantenerumab was studied in PSAPP mice at 7 months (Bohrmann et al., 2012). After a single i.v. injection, plasma levels of Gantenerumab rapidly fell over one week while brain levels rose within this time and persisted at high levels for over 2 months indicating effective penetration into the brain (Bohrmann et al., 2012).

Gantenerumab did not affect plasma levels of Aβ, but was found associated with amyloid plaques as early as 3 days (Bohrmann et al., 2012). A 36–70% reduction in Aβ plaques was observed in PSAPP mice after 5 months of weekly Gantunerumab injections. Gantunerumab treatment had a greater effect on reducing smaller plaques (<400 μm^2^) and preventing plaque formation compared to vehicle treated mice. This long term treatment did not cause inflammation, exacerbate CAA or induce microhaemorrhage (Bohrmann et al., 2012). However, direct injection of Gantenerumab into the hippocampus of APP Tg2576 mice showed a small non-significant increase in pro-inflammatory cytokines (IL1β and TNF-α) after 7 days (Fuller et al., 2015).

Another study with a long term treatment regime (weekly i.v. injections for 4 months), showed that Gantenerumab significantly reduced the amount of Aβ42 but not Aβ40 in the brain of mice with the London APP mutation (Jacobsen et al., 2014). These were old mice (13–17 months) treated 4–6 months after the onset of amyloid accumulation and starting to develop CAA (Jacobsen et al., 2014). Immunohistochemistry analysis showed that Gantenerumab treatment reduced both the percentage area covered by amyloid and the plaque number approximating baseline levels in cortex and, to a lesser extent, the hippocampus (Jacobsen et al., 2014). There was no significant effects on CSF Aβ40 or Aβ42 levels after 4 months of treatment (Jacobsen et al., 2014), however, lack of baseline measures in this study also makes interpretation of CSF levels difficult to evaluate.

The mechanism of Gantenerumab induced amyloid clearance is thought to involve microglial phagocytosis (Bohrmann et al., 2012; Ostrowitzki et al., 2012). This is based on *ex vivo* studies using primary human microglial cells co-incubated with sections of AD brain tissue that have been pre-treated with Gantenerumab (Bohrmann et al., 2012; Ostrowitzki et al., 2012). Double immuno-labelling for Aβ and Gantenerumab show cellular uptake by microglia and a dose-dependent decrease in plaque load (Bohrmann et al., 2012; Ostrowitzki et al., 2012). Very few studies examined the effect of Gantenerumab on cognition in mice. No improvement in the MWM test was seen in PS2APP mice after 5 months of treatment, however, this study was compromised by lack of learning in Wt and control animals (Bohrmann et al., 2012).

##### Clinical Trials

Hoffmann La-Roche, Chugai Pharma, and Washington University School of Medicine sponsored four clinical trials of Gantenerumab. A phase 1 PET study in 18 patients with mild-moderate AD demonstrated the safety and potential efficacy of Gantenerumab in clearing amyloid. Gantenerumab was administered at 60 or 200 mg monthly for 7 months and showed a dose-dependent reduction in brain amyloid in [^11^C] PIB-PET scans as well as a decrease in SUVR from baseline with the higher dose. Despite variability in amyloid reduction between patients, with one case having no amyloid reduction, brain regions with highest decrease in SUVR corresponded to areas with high Fluid-Attenuated Inversion Recovery (FLAIR) in MRI scans. The decreases in amyloid occurred after 2 months and persisted to the final 8 month time point. While the treatment was overall well tolerated, two patients that were APOE4 homozygous receiving the 200 mg dose experienced microhaemorrhage and VE which resolved after discontinuation of dosing (Ostrowitzki et al., 2012).

The effect of Gantenerumab on Aβ reduction led to two phase 3 trials, SCarlet RoAD and Marguerite RoAD. SCarlet RoAD was a 2 year study in prodromal AD that was stopped early for futility. Patients were diagnosed based on ADR, FCSRT, MMSE scores, and MRI and CSF Aβ consistent with AD (Lasser et al., 2016). Patients received s.c. injections of 105 mg or 225 mg every 4 weeks. Gantenerumab dose dependently reduced brain amyloid in PET imaging. Amyloid reduction occurred mainly in the first 60 weeks for the 225 mg dose. In contrast to the high percentage amyloid reduction observed in mouse studies, Gantenerumab resulted in a very modest 6% reduction at higher doses and only transient reduction at lower dosages (Ostrowitzki et al., 2017). CSF tau and p-tau levels also decreased in a dose and time dependent manner with change from baseline reaching significant levels at week 104. No change in CSF Aβ_1__–__4__2_ or brain volume as observed with MRI was present compared to placebo. ARIA-E increased with dose and genotype (APOE4) and was 33% greater in 225 mg dosage compared to placebo (Ostrowitzki et al., 2017). Similarly, ARIA-H increased by 7–27% with Gantenerumab treatment and APOE4 genotype, but this was not dependent on dose (Ostrowitzki et al., 2017).

The effect of Gantenerumab on cognitive decline showed no change after 2 years using CDR-SB as the primary endpoint and ADAS-Cog13 and MMSE as secondary measures (Ostrowitzki et al., 2017). Changes in ADAS-Cog13 scores were smaller (0.3–0.6) than with previous studies with Solanezumab (0.8). However, secondary analysis of fast progressing (APOE4 carriers) and slow progressing AD subgroups revealed a dose-dependent improvement in ADAS-Cog13 and MMSE in the slow progressing subgroup (Ostrowitzki et al., 2017). This study was stopped early based on futility analysis but the potential effects of Gantenerumab led to Marguerite RoAD phase 3 trial to incorporate higher doses.

The lack of effect of Gantenerumab on AD progression may be due to restricted doses used to avoid adverse events. For this reason, both of these phase 3 trials were converted to open label extension studies to assess higher doses of Gantenerumab. This involved 6 titration schedules (over 2–6 months) to which patients were assigned with target dose of 1,200 mg (Gregory et al., 2018). In contrast to the core studies, the extension obtained a significant reduction in amyloid burden from extension baseline to week 52, measured by florbetapir PET analysis (Gregory et al., 2018). Mean change in SUVR units were up to 3 times greater than the change seen in SCarlet RoAD, with one third of patients obtaining below threshold PET SUVR signals (Gregory et al., 2018).

Greater effects of Gantenerumab on imaging biomarkers with higher doses has informed ongoing phase 3 trials sponsored by Hoffmann La-Roche and MorphoSys called Graduate 1 and Graduate 2. These studies are enrolling patients with early AD and confirmed AD pathology and aim to administer doses up to 5 times that of Marguerite and SCarlet RoAD studies. Finally, Gantenerumab is also being studied as part of the Dominantly Inherited Alzheimer Network Trial (DIAN-TU trial). This is a worldwide clinical study evaluating potential disease modifying treatments in individuals at risk for or with early-onset AD caused by a genetic mutation. The trial is being run by Washington University School of Medicine at 26 sites across United States, Canada, Australia and Europe aiming to be completed by 2023.

#### Biogen: Aducanumab

Aducanumab (BIIB037) was developed by Neuroimmune and Biogen (Patent: WO2014089500A1). Neuroimmune established a Reverse Translational Medicine (RTM) platform to isolate recombinant human anti-Aβ antibodies from the B-cell library of healthy elderly patients with no cognitive impairment. Aducanumab is a recombinant human monoclonal antibody derived from an endogenous antibody (Ferrero et al., 2016).

##### Preclinical Studies in Mice

Aducanumab, administered as single i.p. injection of 30 mg/kg, bound to parenchymal Aβ in the brains of 22 month TG2576 mice, with less prominent binding to vascular Aβ (Sevigny et al., 2016). This dose did not affect plasma or brain Aβ levels, which is expected as Aducanumab does not bind monomeric Aβ. Repeated weekly doses of ^ch^Aducanumab, a murine analogue, reduced brain Aβ up to 70%, including oligomeric and fibrillar Aβ, in a dose-dependent manner. Histological staining revealed a reduction in plaque number and volume, but not in vascular Aβ from either the cortex or hippocampus. The clearance of Aβ was associated with recruitment of Iba1 positive microglia, suggesting a possible microglia-mediated clearance (Sevigny et al., 2016).

##### Clinical Trial

Aducanumab completed four phase 1 studies (Ferrero et al., 2016) and an extension study (PRIME) (Sevigny et al., 2016). PRIME enrolled mild or prodromal AD patients with number of adverse events as primary outcome. Participants received monthly infusions of placebo or 1, 3, 6, or 10 mg/kg Aducanumab for 1 year. There were significant dose-dependent reductions in PET-imaged amyloid in all affected brain regions after 54 weeks in the 3–10 mg/kg groups, with no differences between prodromal and mild AD, or between APOE4 carriers and non-carriers. Three participants developed transient anti-Aducanumab antibodies which had no apparent effect on safety or pharmacokinetics of Aducanumab. Fifty percent of patients given the highest dose developed ARIA-E (Ferrero et al., 2016; Sevigny et al., 2016). However, a dose-dependent trend in slowing of cognitive decline was observed in CDR-SB and MMSE scores after 54 weeks. The extension trial included all participants given Aducanumab but was halted early when futility analysis was conducted on phase 3 trial data.

The promising phase 1 data led to two phase 3 trials (ENGAGE and EMERGE) including over 1630 participants with MCI or early-stage AD with confirmed pathology. The trials investigating the efficacy and safety of high and low dose of Aducanumab compared to placebo for 78 weeks with long-term extension.

Futility analysis of pooled data from the ENGAGE and EMERGE by an independent group found that Aducanumab was unlikely to meet primary endpoints and both trials were halted in March 2019. However, re-analysis of the full data set from EMERGE by Biogen revealed patients in the high dose group showed evidence of slowed cognitive decline compared to placebo, with a 22% decrease in change of CDR-SOB at 78 weeks (Cummings et al., 2021). Aducanumab trial data was submitted to the U.S Food and Drug Administration (FDA) for marketing approval, however, the committee has recommended further studies as supporting evidence to conclude its efficacy (Knopman et al., 2020; Alexander et al., 2021; Cummings et al., 2021; Fillit and Green, 2021). Since then, on June 7th 2021 the FDA has approved the use of Aducanumab in United States under the Accelerated Approval Pathway.

#### Sanofi: SAR-228810

SAR-228810 is a humanised monoclonal antibody that, like Crenezumab, is engineered into an IgG4 Fc domain. Two amino acid substitutions were introduced at S241P and L248E to reduce effector function and the potential risk of ARIA. SAR-228810 is specific for soluble protofibrillar and fibrillary Aβ, and not monomers (Pradier et al., 2018).

##### Preclinical Studies in Mice

SAR-255952 is the murine version of SAR-228810. SAR-255952 is an aglycosylated IgG1 antibody that was designed based on 13C3 antibody which detects soluble Aβ protofibrils (Schupf et al., 2008; Pradier et al., 2018). Glycosylation of SAR-255952 is intended to limit effector function and proinflammatory response.

3.5 month old APP/PS1 mice were administered weekly i.p. injections of 10 mg/kg SAR-255952. Histological examination of mouse brains 5 months after immunotherapy confirmed that SAR-255952 entered the brain and bound to plaques. Plaque load decreased after treatment by 24% by MRI and 33% by immunohistochemistry (Santin et al., 2016).

Ascending dose study in APPSL mice showed that a minimal dose of 3 mg/kg/week for 20 weeks was sufficient to reduce Aβ plaque accumulation (Pradier et al., 2018). Immunohistochemistry showed a dose dependent decrease in Aβ load in the cortex and hippocampus with a 78–80% reduction accompanied by reduction in inflammatory marker Cystatin-F and preservation of synaptic function (Pradier et al., 2018). Similar effects on Aβ and Cystatin-F reduction were observed when the humanised SAR-228810 was administered to immunotolerised APPSL mice (Pradier et al., 2018). In contrast to 3D6, SAR-255952 did not increase microhaemorrhage or induce vascular changes even when administered i.v. at high (50 mg/kg) doses in aged APPPSL mice (Pradier et al., 2018). No behavioural tests have been reported for SAR-255952.

##### Clinical Trial

SAR-228810 has completed phase 1 trial testing six ascending doses in 48 mild-moderate AD subjects. SAR-228810 was administered via the i.v. or s.c. route up to 4 infusions over a 10-month period. Results from this trial have not been reported yet.

#### Pfizer: Ponezumab

Ponezumab (PF-04360365) was first developed by Rinat Neuroscience. It is a humanised IgG2 monoclonal antibody directed toward amino acids 33–40 in the c terminus of Aβ40, and not Aβ42 (La Porte et al., 2012). The Ponezumab IgG2 antibody contains two mutations in the Fc region (IgG2δa) to eliminate effector function, such that the hypothesised mechanism of Aβ clearance is via a “peripheral sink” mechanism in which plasma antibodies reduce CSF Aβ, rather than the immune-mediated clearance of other immunotherapies(La Porte et al., 2012).

##### Preclinical Studies in Mice

Ascending dose study in 200 Tg2576 mice aged 16–19 months demonstrated a dose dependent increase in plasma Aβ levels. There was no increase in microhaemorrhage or vasogenic edema compared to vehicle treated mice up to 6 months of treatment at 100 mg/kg (Freeman et al., 2012a). In a separate study, PSAPP mice (5 months) were administered weekly i.p. injections of 10 mg/kg Ponezumab for 6 months (Bales et al., 2016). Aβ40 positive leptomeningeal and parenchymal blood vessels were significantly reduced by approximately 50% without increased incidence of microhaemorrhage (Bales et al., 2016). Reverse microdialysis showed a significant increase in ISF Aβ40 after a single dose of Ponezumab compared to untreated mice or young mice that do not have plaques (Bales et al., 2016). Similar results were obtained in plaque bearing APP/PS1dE9 mice and suggests mobilisation of Aβ plaques after immunotherapy. The vasomotor response to acetylcholine was additionally rescued after acute Ponezumab immunotherapy (Bales et al., 2016). No behavioural studies have been reported with Ponezumab.

Ponezumab demonstrated safety in toxicology assessments in cynomolgus monkeys. Ascending doses from 10 to 100 mg/kg were administered in 27 i.v. injections 10 days apart (Freeman et al., 2012b). Ponezumab immunotherapy resulted in increased Aβ40 plasma levels compared to vehicle. Ponezumab could be detected in the CSF.

##### Clinical Trial

Ponezumab completed five phase 1 trials and three phase 2 trials in mild-moderate AD patients. Single doses ranging between 0.1 and 10 mg/kg were investigated (Burstein et al., 2013; Landen et al., 2013; Miyoshi et al., 2013). There was a dose dependent increase in plasma Aβ levels after 2 h infusion of Ponezumab and no evidence of microhaemorrhage by MRI (Miyoshi et al., 2013). CSF Aβ was found to increase 38% from baseline with the 10 mg/kg dose (Landen et al., 2013).

Mild-moderate AD was diagnosed based on MMSE scores, Diagnostic and Statistical Manual of Mental Disorders, and NINCDS-ADRDA. Patients received 10 infusions, 60 days apart, of one of five doses between 0.1 and 8.5 mg/kg or placebo with a 6-month follow-up period. Ponezumab was detected in the CSF at less than 1% of plasma concentrations. A dose dependent increase in Aβ40, not Aβ42, was detected in plasma but not CSF. No effect was observed on cognitive outcome in ADAS-Cog or DAD scores, brain volume, or CSF tau levels. There was a lower incidence of TRAE compared to placebo including ARIA-H and cerebral microhaemorrhage (Landen et al., 2017b). Similar results in cognitive scores, plasma Aβ, CSF penetration and biomarker levels were observed in a separate phase 2 study. AD patients received either 10 mg/kg dose of Ponezumab every 3 months, or an initial 10 mg/kg dose followed by monthly 7.5 mg/kg infusions, for 1 year. There was no change from baseline in brain amyloid measured by PET at month 13 (Landen et al., 2017a).

Another phase 2 study was conducted in patients with probable CAA. Three doses of Ponezumab were administered at 10 mg/kg followed by 7.5 mg/kg 30 days apart. Cerebral microhaemorrhage was approximately 20% higher with Ponezumab than the placebo group. A trend toward reduced cerebrovascular activity, measured by blood oxygenation level dependent fMRI, was recorded with Ponezumab immunotherapy, but this was a 2 month study in which long term effects were not investigated (Leurent et al., 2019). Pfizer discontinued Ponezumab in 2016.

#### AstraZeneca: MEDI-1814

MEDI-1814 was originally developed by MedImmune and was taken over by AstraZeneca and Eli Lilly. It targets the c terminus of monomeric and oligomeric Aβ_2__9__–__4__2_ (Valera et al., 2016). It is a fully human IgG1λ monoclonal antibody with three mutations within the Fc region to reduce effector function and activation of microglia (Jing et al., 2016; Valera et al., 2016).

##### Preclinical Studies in Mice

MEDI-1814 demonstrated > 1,000-fold selectivity for Aβ42 over Aβ40. When administered to V717I transgenic mice, naïve rats and cynomolgus monkeys, MEDI-1814 reduced CSF Aβ42 up to 90% (Billinton et al., 2017).

##### Clinical Trial

MEDI-1814 has completed one phase 1 multiple ascending dose study in mild-moderate AD. Patients received three i.v. doses from 25 to 1,800 mg or s.c doses at 200 mg (4 weeks apart). MEDI-1814 was detected in CSF and a dose dependent increase in total CSF Aβ42, and not Aβ40, was observed. There was no incidence of ARIA (Ostenfeld et al., 2017).

### Active Immunotherapy

#### Cytos Biotechnology: CAD106

CAD106, sponsored by Cytos Biotechnology and Novartis Pharmaceuticals, comprises 350–550 Aβ_1__–__6_ peptide molecules conjugated to a carrier virus like particle (VLP) from *Escherichia coli* RNA bacteriophage Qβ. VLPs have been incorporated in a number of vaccines for infectious disease but CAD106 was the first to introduce this for neurodegenerative disease.

VLP are non-infectious multiprotein structures which have high antigenic similarity to the virus from which they are derived (Chackerian, 2010). The high density of viral proteins enhances antigen-B cell interactions increasing the magnitude of the antibody response. This means VLP can activate a B-cells at lower concentrations without adjuvants (Dintzis and Vogelstein, 1976). VLPs also contain endogenous Th-cell epitopes enabling the formation of memory B-cells. Conjugating target antigens to VLPs can therefore overcome B-cell tolerance to self-peptides like Aβ.

##### Preclinical Studies in Mice

The efficacy of CAD106 was tested in three different APP mouse models as well as in rhesus monkeys (Wiessner et al., 2011). CAD106 was effective at inducing Aβ specific antibodies in both mice and monkeys at a 25 μg dose (Wiessner et al., 2011). Purified antibody from immunised monkeys recognised both Aβ monomers and oligomers (Wiessner et al., 2011). CAD106 induced antibodies were able to neutralise Aβ induced toxicity *in vitro* (Wiessner et al., 2011).

APP Tg mice were given 3 subcutaneous injections with 25 μg CAD106, 25 μg Qβ, 100 μg AB1–42 + Freund’s adjuvant, or PBS as a control and examined for Th1 cell response and Aβ plaque reduction (Wiessner et al., 2011). T-cell activation by CAD106 was assessed in splenocytes 10 days after final immunisation. In mice immunised with Aβ_1__–__4__2_, stimulation of splenocytes with Aβ_1__–__4__0_ and Aβ_6__–__2__0_ peptides, which contain T-cell epitopes, resulted in a 3–4-fold increase in IFN-γ secreting T-cells (indicative of a Th1 cell response). No effect was observed in CAD106 immunised mice. Instead, T-cell help was provided by Qβ reactive T-cells.

**FIGURE 2:**
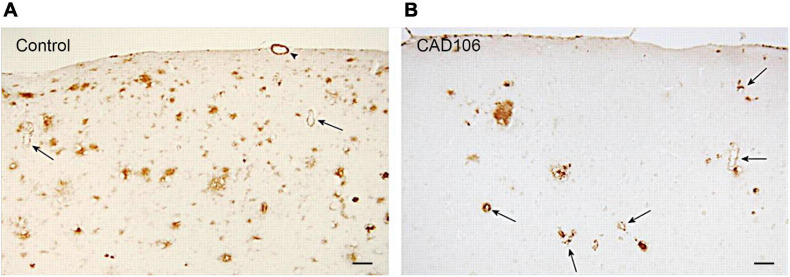
Aβ deposition in the neocortex of APP24 mice after **(A)** PBS or **(B)** CAD106 treatment. Reduction of plaques and worsening of CAA is seen after immunisation (arrows). Scale bar = 100 μm. Reproduced with permission from Wiessner et al. (2011).

To test the preventative effects of CAD106 on development of AD pathology, APP24 mice were immunised every 4 weeks before neocortical Aβ accumulation (7.5 month), 1 month after onset of Aβ pathology and with advanced plaque deposition (13.5–21.5 month). CAD106 had similar effects on plaque reduction (up to 80% in the hippocampus) 8–10 months after treatment (Wiessner et al., 2011). Plaque reduction became less effective with age as pathology advanced with only 17–68% less plaque coverage in the hippocampus. However, a reliable comparison cannot be made in this study due to different treatment time frames being shorter (4–6 months) in the aged mice compared to young mice (10 months) which may partially account for reduced effect. Similar observations were made in a different APP23 mouse model with reduced effect of vaccination with Aβ load. In both APP mice, the reduction was mainly in Aβ42 with little effect on Aβ40. Not surprisingly, the reduction in Aβ plaques with CAD106 treatment reciprocated in increased vascular Aβ42 (not Aβ40) as shown in Figure 2 which is consistent with observations in AN1792 studies (Wiessner et al., 2011). Despite this, there was no increase in microhaemorrhage with CAD106. No behavioural studies were reported for CAD106 so the functional outcome of CAD106 immunotherapy was not determined.

##### Clinical Trials

CAD106 was tested in a 52 week, phase 1 trial in mild-moderate AD, based on Diagnostic and Statistical Manual of Mental Disorders version IV, and NINCDS-ADRDA (Winblad et al., 2012). 58 patients were given three s.c. injections of either 50 or 150 μg CAD106, or placebo. Sixty seven to eighty two percent of patients obtained adequate antibody titres (Winblad et al., 2012).

A phase 2b 90 week study investigated the effect of two adjuvants Alum and MF59 which showed no difference on production of antibodies. Antibodies purified from CAD106-immunised patients bound to Aβ plaques in human AD brain sections and correlated with patient antibody titres (Winblad et al., 2014; Vandenberghe et al., 2017). Patients received three s.c. or i.m. injections 150 μg of CAD106 and a further four injections in an extension study. I.m. administration resulted in higher Aβ titres after the first three injections compared to s.c. injections. The plasma Aβ_1__–__4__0_ levels increased upon repeated injections suggesting translocation of Aβ from the brain into the bloodstream. MRI showed no difference in cerebral atrophy between CAD106 and placebo. CAD106 did not affect ADAS-Cog scores for cognitive decline in AD patients. Similar to the phase 1 study, after 52 weeks no change in CSF biomarkers was observed, however, a decrease in CSF p-Tau levels occurred in extension studies. T-cell responses were established by measuring the change in the number of plasma cells secreting IFNγ which only occurred after stimulation with Qβ but not Aβ.

The CAD106 vaccine was generally well tolerated with one patient developing subarachnoid haemorrhage followed by an intracerebral haemorrhage (Farlow et al., 2015). No meningoencephalitis, CNS inflammation, autoimmune disease or ARIA-E were reported, however, three cases of ARIA-H occurred with CAD106 treatment (Farlow et al., 2015).

#### Elan/Wyeth: ACC-001

ACC-001 (Vanutide cridificar) is composed of multiple short fragments of Aβ_1__–__7_ conjugated to a non-toxic variant of the carrier protein diphtheria toxin (CRM197) and QS-21 was used as an adjuvant. The N-terminal fragment Aβ_1__–__7_ has been shown to contain a B-cell epitope while avoiding T-cell epitopes. This was the one of the first AD immunisation studies to utilise PET imaging to measure cortical amyloid burden.

##### Preclinical Studies in Mice

Few preclinical studies have been published for AC-001. Immunising non-human primates with ACC-001 + QS-21 produced an anti Aβ-antibody response similar to AN1792 but did not generate Aβ directed T-cell responses (Hagen et al., 2011).

##### Clinical Trials

ACC-001 demonstrated good safety, tolerability and immunogenicity in two phase 2 studies in mild-moderate AD patients with elevated baseline brain amyloid (Ketter et al., 2016; van Dyck et al., 2016). ACC-001 was formulated in QS-21 adjuvant (equivalent to AN1792 formulation) and injected i.m. at 3 or 10 μg on 6 occasions over a period of 18 months, patients were then evaluated for safety for another 6 months. No significant change from baseline in the primary endpoint of fibrillar amyloid burden was observed or CSF P-tau, however, there was a slight dose dependent decrease. ACC-01 was well tolerated with no ARIA-E reported in a cohort of 51 patients (van Dyck et al., 2016). In a larger trial of 92 participants, 6% reported ARIA-E compared to 0% in placebo (Ketter et al., 2016). In this study, the reduction in brain volume, measured by vMRI, was accelerated in the group receiving 10 μg dose, but not the 3 μg dose, with a 4.2 ml/year brain volume loss compared to 1.3 ml/year in the placebo group (Ketter et al., 2016).

Three further phase 2 studies in the EU/US and Japan assessed multiple ascending doses of ACC-01 ranging from 3 to 30 μg with or without QS-21 in over 200 patients with mild-moderate AD (Arai et al., 2015; Pasquier et al., 2016). Firstly, QS-21 was necessary to produce a strong, sustained anti-Aβ antibody response (Arai et al., 2015; Pasquier et al., 2016). Amyloid burden was measured by ^18^F-florbetapir PET imaging. While the decrease in amyloid burden showed a dose dependent trend, no statistically significant difference was observed between treatment groups and placebo. This was accompanied, however, by a significant increase in plasma Aβ_x__–__40_ 12 months after immunisation, indicative of increased clearance into the blood (Pasquier et al., 2016). No difference was observed in cognitive scores, vMRI, or CSF biomarkers between treatment groups and placebo. 0.8% patients reported ARIA-E (Arai et al., 2015; Pasquier et al., 2016). These trials underwent one-year extension study which included 4 additional injections of the vaccine. Treatment-related SAEs occurred in 3.1% (EU/US) and 11.3% (Japan) of subjects. AEs leading to withdrawal from treatment or the study occurred in 8.8% of subjects in the EU/US studies and 15.1% in the Japan study. There was no change in cognition as measured by MMSE in parent or extension study (Hüll et al., 2017). The trials were terminated due to lack of efficacy which may have resulted from the short study duration and insufficient antibody titres.

#### AFFiRiS: AD02

AFFiRiS peptide vaccines are developed using AFFITOPE technology. AD02 was developed using peptide mimicry to produce short “non-self” peptides that resemble the N-terminus of Aβ1–6 and avoid humoral autoimmunity. This is conjugated to KLH and adsorbed to Alum (Schneeberger et al., 2009, 2010). As mentioned previously, MHC II molecules bind to peptides that are 12–15 amino acids in length, therefore by restricting the antigen to 6 amino acids and excluding bona-fide T-cell epitopes avoids activation of antigen-specific autoreactive T-cells. In addition, the short peptide prevents cross-reactivity with APP leading to a more targeted response.

##### Preclinical Studies in Mice

AD02 has been assessed in Tg2576 mice. Mice were given six 30 μg injections of either AD02 or a control peptide, Aβ_1__–__6_ (0.1% Alum), at monthly intervals (Mandler et al., 2015). AD02 showed no cross-reactivity with murine Aβ_1__1__–__4__2_ or APP and had a 3-fold higher preference for fibrillary forms of Aβ compared to oligomers and monomers (Mandler et al., 2015). In Tg2576 mice, the vaccine demonstrated a safe immune response while effectively clearing 70% of insoluble Aβ deposits from the brain, however, no change was observed in the levels of soluble Aβ_1__–__4__0_ or Aβ_1__–__4__2_ (Mandler et al., 2015). Despite the decrease in parenchymal amyloid, CAA and microhaemorrhage in the cortex and hippocampus did not increase after 6 months of treatment (Mandler et al., 2015).

The ability of AD02 to induce T-cell activation was investigated *in vitro* by isolating splenocytes from Wt mice which had received 3 AD02 injections (2 week apart). This showed that AD02 did not activate Aβ specific T-cells, however, T-cell infiltration into the brain was not investigated in these mice (Mandler et al., 2015).

Functional outcome of immunotherapy was assessed for spatial and contextual memory in the Morris water maze (MWM) and contextual fear conditioning (CFC) (Mandler et al., 2015). While no difference in learning capability was found between AD02 treated and control mice, memory retention was improved with AD02 in MWM tests with 42% increase in performance with AD02 treatment. Similarly AD02 treated mice showed significantly improved memory recall in CFC tests (Mandler et al., 2015).

##### Clinical Trials

The safety and tolerability of AD02 was tested in a phase 1 trial in Austria. 24 participants with mild-moderate AD (based on MMSE score and MRI scans) were given four repeated subcutaneous doses of AD02 at monthly intervals. After 1 year, AD02 demonstrated a favourable safety profile with no occurrence of meningoencephalitis.

A phase 2 study was conducted across Europe in patients with early AD (mild plus prodromal AD) to test the safety and immunological activity of AD02 following repeated s.c. administration. Patients were diagnosed based on NINCDS/ADRDA, MMSE score, MRI and CSF biomarkers (p-Tau and reduced Aβ) (Schneeberger et al., 2015). Patients were given 6 injections of either AD02 or Alum over 65 weeks. No difference in cognition or function was observed with AD02 in adapted ADAS-cog and ADCS-ADL tests, respectively (Hendrix et al., 2015; Schneeberger et al., 2015). AD02 did not show any improvement in the progression of AD, as measured by Clinical Dementia Rating Sum of Boxes (CDR-SOB) (O’Bryant et al., 2008). While MRI hippocampal brain volume decreased by similar amounts in all patient groups, the rate of decrease of whole brain volume appeared to be accelerated with higher doses of AD02. The apparent lack of effect may be due to the antibody response being higher against the conjugated KLH (82–93%) than the actual AD02 peptide (69–85%) and aggregated Aβ (31–46%) (O’Bryant et al., 2008). In terms of safety profile, no evidence of meningoencephalitis and ARIA-E were reported and the incidence of micro-haemorrhages and ARIA-H was within the expected range. However, the number of patients with AEs increased with immunisation and led to a 19% drop out (O’Bryant et al., 2008; Schneeberger et al., 2015). Serious AEs increased with Alum concentration and AD02 dose by approximately 5%, however Alum alone had low incidence of SAEs suggesting that the majority were due to AD02 (Schneeberger et al., 2015). Failure to show treatment benefit of AD02 and to reach the desired immune response precluded further development of the vaccine (Schneeberger et al., 2015).

#### United Neuroscience: UB-311

United Neuroscience (recently renamed Vaxxinity) has developed an anti-Aβ vaccine (UB-311) that has enhanced functional antigenicity and immunogenicity based on their UBITh peptide technology.

UB-311 comprises a fully synthetic peptide, in which intrinsic self T-cell epitopes are replaced by foreign un-selective UBITh T helper peptides that are covalently linked to the functional antigenic Aβ peptides (Wang et al., 2007, 2017). Use of foreign T helper peptides increases the immunogenicity of the vaccine and reduces the need for strong adjuvants to elicit an immune response. In addition, the UBITh platform avoids the use of a toxoid carrier, which has been shown to promote immune responses against the carrier protein rather than the antigen. UBITh therefore enhances the B-cell response to specifically produce site-directed antibodies against Aβ (Wang et al., 2007, 2017). Thus the UBITh platform specifically modulates components of the immune system in a way not done before. Pre-clinical studies in immunised Macaques showed no brain swelling, microglial or astrocyte activation, and infiltration of T-cells was not detected (Wang et al., 2007).

##### Clinical Trials

In a Phase I clinical trial in Taiwan, UB-311 demonstrated good safety and tolerability in mild to moderate AD patients. In this study, UB311 has uniquely been found to elicit near a 100% immune response rate, which is not seen in most other vaccines (Wang et al., 2007). Antibodies produced after immunisation demonstrated preferential binding to oligomeric and fibrillar forms of Aβ. UB-311 has entered Phase II clinical trials in mild-moderate AD patients to assess safety/tolerability, immunogenicity and cognitive, functional, global, and neuropsychiatric outcomes (Wang et al., 2007, 2017).

### Insights Gained From Aβ Immunotherapy Studies to Date

Two decades of work, initially in experimental models and subsequently in human clinical trials, attempting to produce a treatment for Alzheimer’s disease has not yet successfully resulted in a fully licenced therapy. Very recently, Aducanumab has been given approval under the FDA’s accelerated approval pathway, requiring follow up studies. However, a considerable amount has been learned. Both active and passive immunotherapies can trigger removal of Aβ plaques in experimental models. Aβ removal by immunotherapy can be translated successfully to humans, as demonstrated initially by post mortem neuropathology and subsequently by amyloid PET imaging. High levels of therapeutic antibody are required in order to ensure effective penetration into the brain.

No improvement in cognitive function has been demonstrated and evidence for slowing of cognitive decline has been limited or absent. In addition to the heterogeneicity in patient selection, the most likely explanation for this limited efficacy would seem to be either that Aβ is not the appropriate target and Aβ accumulation in the brain in AD is an epiphenomenon, or that while Aβ accumulation plays a key role in initiating AD pathology other pathological processes set up self-perpetuating cycles such that removal of Aβ in established AD is ineffective (Boche and Nicoll, 2020). It remains to be seen if Aβ immunotherapy, ideally active vaccination in order to be practicable, can prevent AD if given before disease onset.

There are side effects associated with removing Aβ from the human brain, notably ARIA E and H, which are among the more frequently reported adverse events in anti-Aβ immunotherapy. ARIAs are detected by regular monitoring with MRI scans and have been defined across clinical trials as either “symptomatic” or “asymptomatic” (the latter meaning that MRI findings did not translate into symptomatic effects). Most ARIA are asymptomatic and resolve overtime, but there have been a few reports of symptomatic ARIAs that also resolve overtime. ARIA normally presents clinically with mild symptoms of headache, confusion, and neuropsychiatric symptoms, and is not associated with any significant effect on patient cognition (Sperling et al., 2011). The effects of ARIA have been dealt with for passive immunisation by titrating the dosage, pausing then re-starting dosing or withdrawing therapy altogether (Sperling et al., 2011; Salloway et al., 2014). Risk of ARIA is increased with higher Aβ load, degree of CAA, APOE4 status and dose of immunotherapy (Sperling et al., 2011; VandeVrede et al., 2020). The risks, causes and effects of immunotherapy induced ARIA and recommendations for future clinical trials have been discussed in detail by the Alzheimer’s association research round table workgroup (Sperling et al., 2011).

AD is unusual amongst the neurodegenerative diseases in that there is abnormal accumulation of two proteins, Aβ and tau. There is evidence both from post mortem neuropathology and *in vivo* tau PET imaging that removing Aβ from the brain can ameliorate tau accumulation to some extent (Nicoll et al., 2019). This supports the amyloid cascade hypothesis (Panza et al., 2019), but leaves open the possibility that persistent or progressive tau spread after Aβ immunotherapy-mediated plaque removal is the reason for its modest, at best, benefit. A number of tau-targeting therapies, including immunotherapies, are also under development and being explored in clinical trials as a therapy for AD (Panza et al., 2016; Jadhav et al., 2019; Cummings et al., 2020). A key difference between the animal experiments described above and the human trials is the lack of tau pathology in the mice, so that any dysfunction in the mice can reasonably be ascribed to the Aβ accumulation which is not the case for the human trials. Furthermore, none of the vaccination therapies consider that vascular Aβ accumulates due to its failure of intramural periarterial drainage which needs to be addressed before solubilising plaques.

In retrospect, due to this complexity, it may not have been for the best that AD was the first of the neurodegenerative diseases to be chosen in which to explore immunotherapy. Other neurodegenerative disorders are typically characterised by abnormal accumulation of a single protein and this relative simplicity may make them more tractable. A good example is accumulation of α-synuclein as occurs in Parkinson’s disease, Dementia with Lewy bodies and multiple system atrophy and the current state of immunotherapy targeting this protein is explored below.

## Alpha Synuclein Targeted Immunotherapy

Table 3 summarises the clinical trials in PD.

**TABLE 3:** Summary of Clinical trials for passive and active immunotherapy in Parkinson’s disease.

	Phase I		Phase II		
		
	NCT number (participants)	Study duration	NCT number (participants)	Study Name	Study duration
PRX002	NCT02157714 (64)	2014	NCT03100149 (316)	PASADENA	2017–2026
	NCT02095171 (40)	2014			
BIIB-054	NCT02459886 (66)	2015–2017	NCT03318523 (357)	SPARK	2018–2021
	NCT03716570 (24)	2019–2021			
PD03A	NCT02267434 (36)	2014–216			
	NCT02270489 (30)	2014–2017			
MEDI1341	NCT03272165 (49)	2017–2021			
	NCT04449484 (36)	2020–2022			
UB-312	NCT04075318 (40)	2019–2022			
ABBV-0805	NCT04127695	2020			

### Active Immunotherapy

#### AFFiRiS: PD01A and PD03A

PD03A has been developed using the same AFFITOPE technology described in section “AFFiRiS: AD02” (Affiris, 2018).

*In vitro* studies showed that PD03A-induced antibodies targeted αSyn with a stronger preference for aggregated forms. In several animal models, PD03A generated an immune response against full length αSyn and did not cross-react with βSyn (Affiris, 2018).

The vaccine was tested for safety and tolerability in two phase I trials in patients with MSA or PD under the SYMPATH project (Affiris, 2018). A multicentre study in France tested PD01A and PD03A in patients with MSA/PD and monitored the CSF biomarkers and MRI results over a year. Patients received 5 s.c. injections of placebo, 75 μg PD01A or PD03A at 4 week intervals. PD01A induced antibodies were selective for αSyn oligomers over native αSyn (Volc et al., 2020). Both treatments were well tolerated and induced prolonged anti-αSyn titres, with 89% responder rate for PD01A and 58% for PD03A (Affiris, 2018). Clinical scores did not differ between treatment groups during the course of this study. The PD trial was conducted in Austria where 36 PD patients received 4 s.c. injections of either 15 or 75 μg vaccine every 4 weeks with a booster 24 weeks after the last injection (Affiris, 2018; Meissner et al., 2020). Patients were assessed for levels of dopamine receptors using DAT-SPECT and brain volume by MRI over a 12 month period. A robust immune response that was specific against the peptide moiety of PD01A was observed at each dose and there were no reported SAEs related to the drug (Affiris, 2018; Volc et al., 2020). PD01A immunotherapy resulted in a 51% decrease in oligomeric αSyn in the CSF compared to placebo (Volc et al., 2020).

#### United Neuroscience: UB-312

In addition to their Aβ vaccine, United Neuroscience (Vaxxinity) developed UB-312 to target oligomeric and fibrillary αSyn based on the same UBITh technology. Over 60 B-cell epitopes of αSyn were screened for immunogenicity in guinea pigs from which a short-list of 3 epitopes were investigated for their binding properties (Honig et al., 2018). UB-312-derived antibodies demonstrated strong labelling of disease-specific αSyn inclusions in human post mortem brain samples of PD, DLB and MSA brains and showed strong binding to oligomeric and fibrillar forms of αSyn by slot blot analyses (Nimmo et al., 2020).

##### Clinical Trials

UB-312 entered a two part phase 1 trial in Netherlands to assess safety and tolerability of vaccination in healthy and mild PD patients (Hoehn &Yahr Stage ≤ III). Patients will be subject to 20 weeks of treatment with 24 week follow-up period.

### Passive Immunotherapy

Characteristics of passive immunotherapies are summarised in Table 4.

**TABLE 4:** Characteristics of passive immunotherapy for Parkinson’s disease.

	PRX002	BIIB-054	ABBV-0805	MEDI1341
Epitope	C-terminal	N-terminal	N/A	C-terminal
Specificity	Monomers and oligomers	Olgoimeric and protofibrillar	Oligomeric and protofibrillar	Monomers and oligomers
Route of administration	IV	IV	IV	IV
Dose at latest phase	0.3–30 mg/kg (13 doses 4 w apart)	Single shot of 15 mg/kg or 45 mg/kg		3 doses (4 w apart)
Half-life (days)	10–18	28–35		
CSF αSyn level	No change	No change	N/A	N/A
Primary outcome	MDS-UPDRS week 52	MDS-UPDRS week 52 and 72	TRAE and pharmacokinetics	TRAE, vital signs, elecrocardiogram

*IV, intravenous; TRAE, treatment related adverse event.*

#### AstraZeneca: MEDI-1341

MEDI-1341 is a monoclonal antibody manufactured by AstraZeneca and Takeda Pharmaceuticals. Phage display libraries were screened for high-affinity antibodies directed against human αSyn and MEDI-1341 was selected as the lead antibody directed against the C-terminus of αSyn (Schofield et al., 2019).

##### Preclinical Studies in Mice

MEDI-1341 recognised both soluble monomeric αSyn from control human brains as well as higher molecular weight aggregates from PD brains (Schofield et al., 2019). MEDI-1341 entered the CSF after i.v. administration at 100 mg/kg in rats and resulted in an 81% decrease in free αSyn after 2–24 h. Measurements of αSyn in brain interstitial fluid (ISF) by microdialysis showed a rapid 75% reduction in αSyn after MEDI-1341 therapy (Schofield et al., 2019). Further analysis of MEDI-1341 concentration in the brain showed that approximately 0.4% passes from the plasma into the brain (Schofield et al., 2019). MEDI-1341 treatment also prevented the propagation of αSyn in cell culture and a mouse model of αSyn spreading (Schofield et al., 2019).

##### Clinical Trials

Six different doses of MEDI-1341 are being tested in 36–48 healthy subjects in two phase I trials sponsored by AstraZeneca. Each patient received a 1 h i.v. infusion of MEDI-1341 followed by 13 months observation for adverse events, pharmacokinetics, quantification of α-synuclein in blood and CSF, and detection of anti-drug antibodies in serum.

#### AbbVie: ABBV-0805

ABBV-0805, also known as BAN0805, is a humanised anti-αSyn monoclonal antibody developed by BioArctic and AbbVie. ABBV-0805 is specific for oligomeric and protofibrillar αSyn. The antibody was patented (EP2539366) in 2017 in which it was shown to decrease the level of αSyn fibrils, prevent motor impairment and double the life span of transgenic Parkinson mice. A Phase I trial of ABBV-0805 was withdrawn in 2020 for strategic considerations, no results have been published.

#### Prothena-Roche: PRX002

PRX002 (Prasinezumab) is a monoclonal antibody directed to the C terminal domain of soluble and oligomeric α-Syn.

##### Preclinical Studies in Mice

PRX002 (murine version, 9E4) was tested in two preclinical studies using PDGF-αSyn or Thy1-SNCA/61 mice (Masliah et al., 2011; Games et al., 2014). In both studies 6 month old mice were administered weekly injections of 9E4 at 10 mg/ml and the changes in behaviour and neuropathology were investigated. The 9E4 antibody was selected based on its specificity for 14 KDa monomeric αSyn in Tg-mice but not wt mice. 9E4 successfully crossed the BBB after 3 days and accumulated in the CSF, in neurons and αSyn rich regions of the brain over 30 days (Masliah et al., 2011).

9E4 therapy preserved normal full-length αSyn and reduced the number of neurons with insoluble calpain-cleaved αSyn oligomers in the cortex and hippocampus of PDGF-αSyn mice (Masliah et al., 2011). Studies in thy1-SNCA mice similarly showed that 9E4 reduced the amount of αSyn accumulation in neurons and axons in the cortex and striatum. The number of αSyn positive neurons was not affected which may explain why 9E4 did not prevent loss of dopaminergic neurons. Neuropathological findings were reflected in behavioural tests. Both transgenic mice showed deficient learning ability to find a hidden platform in the MWM test compared to WT controls which was ameliorated with 9E4 treatment (Masliah et al., 2011; Games et al., 2014). Motor function was assessed by pole test and rotarod in PDGF-αSyn mice, and the round beam test in Thy1-SNCA mice. 9E4 therapy improved motor function with performance reaching normal control levels in rotarod and round beam tests (Masliah et al., 2011; Games et al., 2014).

*In vitro* analysis of the mechanism of action of 9E4 suggests that it promoted the intracellular clearance of αSyn by autophagy (Masliah et al., 2011). In B103 cells infected with lentiviral-αSyn, 9E4 exposure blocked the calpain-cleavage site on αSyn that had been secreted into the extracellular milieu and reduced the propagation of αSyn to neighbouring neurons by 60% (Games et al., 2014).

##### Clinical Trials

PRX002 has showed favourable safety and pharmacokinetics in a single-dose and multiple-dose phase 1 trial in healthy and mild PD patients. PRX002 administration at 0.3–30 mg/kg resulted in a dose-dependent reduction in plasma αSyn that lasted up to 4 weeks at the highest dose (Schenk et al., 2017). PD patients received three i.v. injections at 28 day intervals and were monitored up to 16 weeks after the final injection (Jankovic et al., 2018). PRX002 antibodies were detected in the CSF in a dose-dependent manner suggesting entry into the CNS with a serum-CSF ratio of 0.3% (Jankovic et al., 2018). However, there were no significant changes from baseline in free or total αSyn in the CSF. Occurrence of TEAEs were not dose dependent (Jankovic et al., 2018).

PRX002 completed a phase II trial in April 2021 (PASADENA) and the outcome was announced in a recent press release by Prothena. Patients were administered PRX002 every 4 weeks for a year. PRX002 failed to meet its primary outcome of change in Movement Disorder Society-Unified Parkinson’s Disease Rating Scale (MDS-UPDRS) total score after 1 year. However positive changes were noted in some secondary and exploratory measures including a significant reduction in motor function decline by 35%, delayed worsening of motor symptoms (assessed by MDS-UPDRS Part III), better cognitive performance and improved blood flow to the putamen (Prothena.com, 2020). Based on this data Prothena are planning a further Phase-IIb study (PADOVA) in patients with early PD.

#### Biogen: BIIB-054

BIIB054 (Cinpanemab) was selected from B-cell libraries as described in section “Hoffmann-La-Roche: Gantenerumab.” It binds to N-terminal residues 1–10 of αSyn without cross reactivity to β- or γ-synuclein (Weihofen et al., 2019).

##### Preclinical Studies in Mice

The selectivity of BIIB054 for different αSyn species was determined by ITC, surface plasmon resonance and ELISA and confirmed that BIIB054 had 800-fold higher affinity for fibrillar αSyn compared to monomeric αSyn (Weihofen et al., 2019). In addition, murine version of BIIB054 detected αSyn present in PD and DLB tissue homogenates but not in control cases, and bound to αSyn in LBs, LNs and synapses in IHC assays (Weihofen et al., 2019).

The pharmacokinetic properties of BIIB054 were tested in rats and cynomolgus monkeys (Wang et al., 2018). BIIB054 was injected i.v. at 10 mg/kg and serum and CSF were sampled over several days (Wang et al., 2018). The antibodies entered the CNS in proportion to dose with CSF levels peaking between 24 and 72 h (Wang et al., 2018).

BIIB054 treatment reduced behavioural and neuropathological impairments in mice injected with preformed fibrils (PFF). Three month old mice received 2–3 i.p. injections of 30 mg/kg BIIB054 prior to intra-striatal inoculation of 2 μl PFF (at a rate of 0.2 μl/min), then received weekly BIIB0054 injections up to 3 months post PFF inoculation (Weihofen et al., 2019). BIIB054 resulted in a 30% reduction in 6 KDa truncated αSyn after 100 days, and a 20% reduction in Dopamine transporter (DAT) loss after 3 months (Weihofen et al., 2019). BIIB054 improved behavioural impairment in the wire hanging test by 50% and delayed the onset of paralysis by 7 days (Weihofen et al., 2019).

##### Clinical Trials

BIIB054 has completed two Phase I clinical trials to test its safety and pharmacokinetics in healthy participants and mild- moderate PD (Brys et al., 2018). Eighteen PD patients received single i.v. injections of BIIB054 of either 15 or 45 mg/kg. Serum to CSF ratios of BIIB054 were similar to that observed in preclinical studies (0.4%) with a blood half-life of 30 days (Brys et al., 2018). BIIB054 was found to complex with αSyn in the blood plasma suggesting target engagement. Overall the drug was well tolerated with no TEAEs (Brys et al., 2018).

The efficacy of BIIB054 was investigated using the MDS-UPDRS score in a phase 2 ascending dose trial (SPARK). PD patients were administered monthly doses of BIIB054 ranging between 250 and 3,500 mg and placebo over 2 years. Patients receiving placebo transitioned to BIIB054 in the second year of the study. Simulation analysis estimated that these doses would achieve 50 to over 90% target engagement in ISF (Kuchimanchi et al., 2020). Due to failure of BIIB054 to meet its primary and secondary endpoints, Biogen has closed the SPARK trial and halted further development of BIIB054.

## Discussion

Experimental models of PD and AD have successfully been utilised to demonstrate positive effects of various immunotherapies on neuropathological and behavioural outcome measures. αSyn immunotherapies have not progressed as far through the pipeline of clinical trials as those for AD in order to adequately evaluate their efficacy. The lack of efficacy in primary outcome measures of clinical trials indicates that there is a lack of translation from animal models to the humans, highlighted in Table 5.

**TABLE 5:** Comparison between Aβ immunotherapy in animal models and human clinical trials.

		Mice					Clinical trials					
			
Vaccine (mouse version)	Target	Model	Aβ /Plaque reduction	CAA	MiH	Cog	Plaques	CAA	CSF-pTau	vMRI loss	ARIA-E	Cog
AN-1792	Human Aβ_1__–__4__2_ +QS-21	PDAPP	N/A	N/A	N/A	N/A	Decrease	Increase	N/A	N/A	6%	No
CAD-106	Aβ_1__–__6_+bacterio-phage Qβ	APP, APP24, APP23, rhesus monkeys	80%	Increase	No	N/A	Decrease	Increase	No change	N/A	0	N/A
ACC-01	Aβ1–7 +QS-21	Non-human primates	N/A	N/A	N/A	N/A	No change	N/A	No change	No change	0.8–6%	No change
AD-02	fibrillar Aβ1-6 + Alum	Tg2576	70%	No change	No	Yes (MWM, CFC)	N/A	N/A	N/A	Increase	0	No change
Bapi (3D6)	Aβ1–5	PDAPP	86%	Decrease	Increase	No (MWM)	Decrease	N/A	Decrease	No change	15%	No change
Solz (M226)	Soluble Aβ16–20	PDAPP, J20	Variable	N/A	No	Variable	No change with PET and IHC	230% increase	No change	No change	0.5–1.1%	Significant change in ADAS-Cog 11
Cren	Conformation Aβ16–24	hAPP^(V7171)^/PS1, Tg256	Variable	N/A	N/A	N/A	No change	N/A	No change	No change	0.60%	Initial decline
Gant	Conformation Aβ aggregates	PSAPP, APP Tg2576	36–70%	No change	No	No (MWM)	Decrease	N/A	Decrease	No change	18–35%	No change
Don (mE8)	Aβ(p3–42)	PDAPP	53%	No change	No	N/A	78%	N/A	Decrease	No change	25%	32% change in iADRS
Lecan (mAb-158)	Protofibrils	Tg-ArcSwe	40%	N/A	N/A	No (MWM)	80%	N/A	Decrease	No change	10%	Significant change in ADAS-Cog14
Adu	Aβ_3–6_	TG2576	70%	N/A	N/A	N/A	Decrease		Decrease		34–35%	22% change of CDR-SOB
UB-311	Aβ1–14	Macaques			No							

*CAA, cerebral amyloid angiopathy; MiH, microhaemorhage; Cog, Cognition; vMRI, volumetric magnetic resonance imaging; MWM, morris water maze; CFC, contextual fear conditioning; IHC, immunohistochemistry.*

### Benefits and Limitations of Animal Models

Preclinical studies are based mainly on mouse models of disease. The development of mouse models are mainly informed by the neuropathological characteristics of neurodegenerative diseases. They model specific aspects of human neuropathology such as protein deposition, neuroinflammation and neurodegeneration. Consequently, they are good predictors of the effects of immunotherapy on neuropathology and pharmacodynamics markers, and allow assessment of target engagement. Animal models generally involve manipulation of specific genes which helps to establish correlations between specific neuropathological features or protein species and functional deficits.

Mice do not naturally develop neurodegenerative diseases and require genetic manipulation or inoculation of toxic material to induce neuropathological aspects of the disease. Even after genetic manipulation, neuropathology does not recapitulate all the characteristic features of neurodegeneration such as selective neuronal loss or multiple proteinopathies and co-morbidities. With current technology it is not possible to simulate the complex array of inflammatory, metabolic and protein changes that occur simultaneously in neurodegenerative diseases.

### Translation Between Mice and Humans

Mouse studies have reliably and consistently predicted pharmacokinetics, pharmacodynamics and neuropathological outcomes of immunotherapy. Both passive and active immunotherapy in mice predict antibody brain penetration, T-cell response, the extent of amyloid clearance, transient increase in CAA and resulting microhaemorrhage. However, neuropathological findings do not reflect the primary outcome measures of clinical trials, which are based on cognitive scores. Behavioural and functional analysis of immunotherapy in mice is therefore essential and has been neglected in many preclinical studies. In those studies that have included behavioural analysis however, improved cognition in mice has not always clearly translated to humans with only modest effects observed in clinical trials. Clear-cut therapeutic effects demonstrated in mice have not been observed in humans.

### Future Directions

In summary, the need for animal models that replicate most of the neuropathological features seen in patients is important to better translate the outcomes of immunotherapy in animals to humans. In light of this, mouse models are under development to more closely represent the human state of disease and include double and triple transgenic mice to mimic the multiple proteinopathies that occur in humans and more recently, humanised mice.

On the other hand, patient selection has also been an important contribution to the failure of clinical trials, which have showed more progress toward slowing of cognitive decline in early disease stages which may have confounded some initial studies (Mintun et al., 2021). Better understanding of disease progression would help identify possible therapeutic windows for successful intervention relevant to the neurodegenerative disease. Perhaps immunotherapy should be administered much earlier than is currently being done when the brain can still compensate for disease processes. This would require better diagnostic biomarkers that would allow to identify people at risk of developing the disease before the onset of the neurodegenerative process. Importantly, post mortem examination of immunised patients’ brains has not been done systematically, however, it has provided valuable information on translating mouse to human experimentation and should be incorporated in the study design of clinical trials.

## Author Contributions

JTN was primary author of the manuscript. LK, AV, RC, JARN, and J-CD contributed to and reviewed the manuscript. All authors contributed to the article and approved the submitted version.

## Conflict of Interest

AV was employed by Yumanity Therapeutics. J-CD was employed by United Neuroscience. The remaining authors declare that the research was conducted in the absence of any commercial or financial relationships that could be construed as a potential conflict of interest.

## Publisher’s Note

All claims expressed in this article are solely those of the authors and do not necessarily represent those of their affiliated organizations, or those of the publisher, the editors and the reviewers. Any product that may be evaluated in this article, or claim that may be made by its manufacturer, is not guaranteed or endorsed by the publisher.
